# Dissection of signaling pathways regulating TrkB-dependent gephyrin clustering

**DOI:** 10.3389/fnmol.2024.1480820

**Published:** 2024-10-24

**Authors:** Lisa-Sophie Wüstner, Simone Beuter, Martin Kriebel, Hansjürgen Volkmer

**Affiliations:** ^1^NMI Natural and Medical Sciences Institute at the University of Tübingen, Reutlingen, Germany; ^2^International Max Planck Research School, Graduate Training Centre of Neuroscience, University of Tübingen, Tübingen, Germany

**Keywords:** TrkB, gephyrin, iLTP, CaMKII, SHC, PLCγ, GABA, plasticity

## Abstract

**Introduction:**

The TrkB receptor is known for its role in regulating excitatory neuronal plasticity. However, accumulating evidence over the past decade has highlighted the involvement of TrkB in regulating inhibitory synapse stability and plasticity, particularly through regulation of the inhibitory scaffold protein gephyrin, although with contradicting results.

**Methods:**

In this study, we extended on these findings by overexpressing rat TrkB mutants deficient in either Shc-or PLCγ-dependent signaling, as well as a kinase-dead mutant, to dissect the contributions of specific TrkB-dependent signaling pathways to gephyrin clustering.

**Results:**

Our results demonstrate that TrkB signaling is required for gephyrin clustering on the perisomatic area of granule cells in the dentate gyrus *in vivo*. To further investigate, we expressed TrkB wild-type and mutants in hippocampal neurons *in vitro*.

**Discussion:**

Under basal conditions, TrkB-Shc signaling was important for the reduction of gephyrin cluster size, while TrkB-PLCγ signaling accounts for gephyrin clustering specifically at synaptic sites. Concomitant, impaired PLCγ signaling was associated with disinhibition of transduced neurons. Moreover, chemically induced inhibitory long-term potentiation (chem iLTP) depended on TrkB signaling and the activation of both Shc and PLCγ pathways.

**Conclusion:**

Our findings suggest a complex, pathway-specific regulation of TrkB-dependent gephyrin clustering, both under basal conditions and during chem iLTP.

## 1 Introduction

The GABAergic system is the primary inhibitory system essential for the proper functioning of the central nervous system (CNS). It is crucial for the formation of learning and memory, cognition, long-term potentiation (LTP) and maintaining an appropriate excitatory/inhibitory (E/I) neurotransmission balance. Abnormalities in this system are linked to neuropsychiatric disorders such as Autism Spectrum Disorder (ASD) (Zhang et al., 2021) and schizophrenia (Gao and Penzes, 2015) but also in stress-related conditions such as major depressive disorder (MDD) and post-traumatic stress disorder (PTSD) (Luscher et al., 2011).

One of the key proteins responsible for the stabilization and plasticity at inhibitory synapses is the scaffold protein gephyrin (Choii and Ko, 2015; Groeneweg et al., 2018). Gephyrin anchors GABA type A (GABA_A_) receptors, facilitating their clustering at postsynaptic sites (Kasaragod and Schindelin, 2018). Several protein kinases, including various receptor tyrosine kinases (RTKs) such as TrkB, TrkC, FGFR1, EphA7, and c-Met, as well as members of the MAPK pathway and the PI3K-Akt–mTOR pathway, GSK3β and CDK5 have been identified as regulators of gephyrin clustering (Beuter et al., 2016; Jeckel et al., 2021; Kalbouneh et al., 2014; Tyagarajan et al., 2013; Wuchter et al., 2012). Likewise, gephyrin phosphorylation through CaMKII favors gephyrin clustering and may contribute to homeostatic regulation (Petrini et al., 2014; Flores et al., 2015).

Out of the RTKs, TrkB received attention due its contribution to neuronal plasticity while it is also involved in gephyrin clustering (Minichiello et al., 2002). The TrkB ligand brain-derived neurotrophic factor (Bdnf) modulates activity-dependent glutamatergic synaptic plasticity by regulating spine density and morphology (Squinto et al., 1991; Ji et al., 2010; Leal et al., 2014; Yoshii and Constantine-Paton, 2014; Zagrebelsky and Korte, 2014). At inhibitory GABAergic synapses, Bdnf acts differently depending on the duration of Bdnf exposure *in vitro*. Short term protocols favor removal of gephyrin clusters while long-term exposure shows an increase (Mou et al., 2013). Similarly, reports on the function of the protein kinases Erk are controversial. Degradation of gephyrin relies on post-translational modification by Erk1/2 and calpain**-**dependent degradation (Tyagarajan et al., 2013; Tyagarajan et al., 2013), while Erk activity was reported to be required for gephyrin clustering (Wuchter et al., 2012).

Bdnf-dependent activation induces dimerization and autophosphorylation of the rat TrkB at two different tyrosine residues at positions 515 and 816 in the cytosolic domain. Phosphotyrosine-515 serves as a recognition site for the adaptor Shc which recruits additional factors for the induction of both MAPK and PI3K-Akt signaling (Atwal et al., 2000; Gu et al., 2000). PLCγ interaction at Phosphotyrosine-816 and subsequent activation triggers the release of calcium from internal stores and may thereby activate CaMKII (Minichiello, 2009; Moya-Alvarado et al., 2024; Leal et al., 2015; Zeng et al., 2010). By specifically mutating tyrosine residues the activation of the cognate downstream effectors can be efficiently inhibited (Minichiello et al., 2002).

Here, we applied long-term overexpression of TrkB mutants with deficiency in Shc or PLCγ binding as well as a kinase dead (KD) mutant to dissect TrkB-dependent signaling pathways in their role for gephyrin clustering in homeostasis. Our results suggest complex regulation of gephyrin clustering during basal conditions and chemically induced inhibitory long-term potentiation (chem iLTP) by diverse actions of TrkB-dependent signaling pathways depending on the experimental paradigm.

## 2 Materials and methods

### 2.1 Construction of TrkB mutants

The pEGFP-N1-TrkB plasmid for expression of rat TrkB [a gift from Rosalind Segal (Addgene plasmid # 32500; RRID: Addgene_32500)]^1^ was mutated by site-directed mutagenesis (QuikChange Kit, Agilent, Santa Clara, CA) to give yield to mutants deficient in Shc or PLCγ interaction (Minichiello et al., 2002) or in ATP-binding (McCarty and Feinstein, 1998). Respective primers (Supplementary Table S1) introduced point mutations in base triplets encoding Y515 or Y816 to generate TrkB Y515F (TrkB SHC-), TrkB Y816F (TrkB PLC-) or the kinase-dead mutant (TrkB K571A, TrkB KD). All mutations were subsequently verified by Sanger sequencing.

### 2.2 Western blot

Wildtype (WT) TrkB or TrkB mutants were transfected into HEK293 and 3 T3 cells cultured in 6-well plates to 70% confluency alongside a control plasmid (pEGFP-N1, Takara Bio USA, Inc., San Jose, CA) using Lipofectamine 2000 (Thermo Fisher Scientific, Waltham, MA). After further incubation for 24 h, cells were subjected to FBS starvation for 24 h for the purpose of cell cycle synchronization and to minimize the activation of signaling pathways unrelated to Bdnf stimulation, e.g., through growth factors, hormones and nutrients contained in FBS. Prior to lysis, the cells were either stimulated with 100 ng/mL Bdnf in 0.1% BSA/PBS (PeproTec, Thermo Fisher Scientific, Waltham, MA) for 30 min or only with 0.1% BSA/PBS as a control. After a single wash with PBS, the cells were harvested and lysed in RIPA buffer (Thermo Fisher Scientific, Waltham, MA) supplemented with cOmplete™ mini protease inhibitors (Roche, Rotkreuz, Switzerland) and Pierce™ Phosphatase Inhibitors (Thermo Fisher Scientific, Waltham, MA) for 30 min on ice. Protein concentrations were determined using the Pierce™ BCA Protein Assay Kit (Thermo Fisher Scientific, Waltham, MA) according to the manufacturer’s protocol.

Cell lysates (30 μg of protein per lane) were separated by SDS-PAGE (NuPAGE 4–12% Bis-Tris gels, Thermo Fisher Scientific, Waltham, MA). After transfer, PVDF membranes were incubated with the following primary antibodies: rabbit anti-phospho-TrkA (Tyr674/675)/TrkB (Tyr706/707) (#4621), mouse anti-phospho-p44/42 MAPK (Thr202/Tyr204) (#9106), rabbit anti-p44/42 MAPK (#9102), rabbit anti-phospho-PLCγ1 (#2821) and rabbit anti-PLCγ1 (#2822) from Cell Signaling Technology, Danvers, MA and goat anti-TrkB (AF1494) from R&D Systems, Minneapolis, MN.

Detection was achieved by secondary antibodies which were either fluorescently labeled (for phospho-MAPK and MAPK detection) or HRP-labeled (for phospho-TrkB, TrkB, phospho-PLCγ1 and PLCγ1). Fluorescent signals were acquired using a LI-COR Imager (LI-COR Biosciences, Lincoln, NE), while ECL signals were detected with SignalFire™ Plus ECL Reagent (Cell Signal Technology, Danvers, MA) subsequently acquired with an ImageQuant™ LAS-400 mini (GE Healthcare, Freiburg, Germany). To quantify the relative protein bands, densiometric analysis was performed with Image Lab (Bio-Rad Laboratories, Feldkirchen, Germany).

### 2.3 Construction of lentiviral expression vectors

miRNAs for the knockdown of TrkB (mi1973, mi162, or miTrkB), were transferred into pLenti04C/SEW (Kriebel et al., 2020) through Gateway^®^-recombination (Life Technologies, Waltham, MA). The vector expresses EGFP under the control of a synapsin protein and miRNA under the control of a CaMKII promoter. Sequences targeted by miRNAs can be found in Supplementary Table S2.

Based on a previously described lentiviral vector [pLenti4CaMKII_s/V5-DEST (Kriebel et al., 2020)], TrkB expression vectors were constructed. TrkB WT, TrkB Shc-, TrkB PLC-, and TrkB KD fused to EGFP are driven by a functional mouse *α*-CaMKII promotor fragment. For vector details see Supplementary Figure S3. Lentiviral suspensions were produced in HEK239FT cells under serum-free conditions, purified as previously described (Kriebel et al., 2020) and titred with the Lenti-X qRT-PCR titration Kit (Takara Bio USA, Inc., San Jose, CA).

### 2.4 Preparation, culture, and treatment of primary neurons

Adult pregnant female Sprague–Dawley rats were supplied by Janvier Labs, Le Genest-Saint-Isle, France. The housing of animals and submission to surgical procedures were performed in accordance with the European Union recommendations for the care and use of laboratory animals (2010/63/EU) and were approved by the regional authority (Regierungspräsidium Tübingen). Primary hippocampal neurons were isolated from E18 rat embryos and cultured as described earlier (Kriebel et al., 2020). Cells were seeded on polyethyleneimine (PEI)-coated 96-well μCLEAR^®^ plates (Greiner Bio-One, Frickenhausen, Germany) at a density of 4 × 10^4^ cells/well. On day 3 *in vitro* (DIV3), the cells were lentivirally transduced with 2 × 10^6^ viral copies (VCs)/well.

For chemical induction of inhibitory long-term potentiation (chem-iLTP), experimental procedures were adapted from (Petrini et al., 2014). At DIV14, the cells were washed and treated with 145 mM NaCl, 2 mM KCl, 2 mM CaCl, 2 mM MgCl, 10 mM Glucose, 10 mM HEPES (Pennacchietti et al., 2017) supplemented with 20 μM NMDA and 10 μM CNQX (MedChemExpress, Monmouth Junction, NJ) for 2 min, followed by a recovery period in the treatment solution for 18 min. To inhibit MEK1, CaMKII or TrkB, neurons were treated with either 50 μM PD98059, 3 μM KN-62, 1 μM Cyclotraxin B (all from MedChemExpress, Monmouth Junction, NJ) or DMSO (CTR) for 30 min in culture medium before induction of chem iLTP as described. All inhibitors and DMSO were present during the stimulation and recovery period.

### 2.5 Stereotaxic injection of lentiviral suspensions

Adult female Sprague–Dawley rats (250 g at the time of surgery) were used for stereotaxic injection of lentiviral suspensions. The animals were deeply anesthetized with 2–5% isoflurane/oxygen, followed by s.c. injection of metamizole (50 mg/kg) for intraoperative analgesia at least 30 min before the start of surgical procedures. Bilateral injections of 2.5 μL of lentiviral suspensions into the dorsal dentate gyrus (AP: −2.9 mm, ML: ±2.5 mm, DV: −4.3 mm; all coordinates relative to Bregma) were conducted using a Lab Standard™ Stereotaxic Instrument (Stoelting, Wood Dale, IL) connected to a 701 RN Hamilton syringe (10 μL, 30 gauge, pst 4; CS-Chromatographie Service, Langerwehe, Germany). Injection speed was set to 0.2 μL/min. To assure sufficient postoperative analgesia, 2 mg/kg meloxicam was administered by s.c. injection at the end of surgical procedures and during surgical follow-up care.

For fixation of brain tissue, animals were deeply anesthetized by ketamine (100 mg/kg i.p.) and xylazine (10 mg/kg i.p.) application and transcardially perfused with 100 mL of PBS followed by 250 mL of freshly prepared 4% paraformaldehyde/PBS 14 days after injection. Brain tissue was collected and additionally fixed in 4% paraformaldehyde/PBS at 4°C for 60 min.

### 2.6 Immunohistochemistry and immunocytochemistry

Perfusion-fixated brains were washed in PBS and cut into 50 μm slices (Vibratome VT1000S, Leica Microsystems, Wetzlar Germany). After permeabilization (0.5% Triton X-100), slices were blocked by 1x BMB blocking reagent (Roche, Rotkreuz, Switzerland) for 2 h at room temperature. Further processing of brain slices and immunocytochemical staining was performed as described, previously (Beuter et al., 2016). Specimens were mounted on microscopic slides using Dako Fluorescent Mounting medium (Agilent, Santa Clara, CA).

Untreated or treated primary rat neurons were fixed at DIV14 in 4% PFA/PBS for 15 min at room temperature. After blocking and permeabilization for 30 min at room temperature in 0.1% Triton X-100/PBS containing 1 X BMB blocking reagent (Roche, Rotkreuz, Switzerland), cells were incubated at 4°C with primary antibodies diluted in the blocking solution overnight. Subsequently, secondary antibodies diluted in blocking solution were added for 2 h at room temperature. Nuclei were stained using Hoechst Dye 33,258 (Merck, Rahway, NJ). The following primary antibodies were used: polyclonal rabbit anti-TrkB (Ab6180, 1:50) from Abcam, Cambridge, UK, polyclonal chicken anti-MAP2 antibody (PA1-10005, 1:2500) and polyclonal rabbit anti-GABA (A2052, 1:500) from ThermoFisher, Waltham, MA, mouse anti-VGSC antibody (S8809, 1:100) from Sigma-Aldrich, St. Louis, MO and monoclonal mouse anti-gephyrin (#147021, 1:500), polyclonal rabbit anti-VGat (#131003, 1:500), monoclonal rabbit anti-Psd95 (#124011, 1:500) and monoclonal mouse anti-VGlut1 (#135511, 1:400), all from Synaptic Systems, Göttingen, Germany.

### 2.7 Image acquisition

Confocal fluorescence images of tissue samples from *in vivo* experiments were acquired using a Zeiss LSM510 Meta confocal microscope equipped with a 63 × Plan-Apochromat oil immersion objective (NA 1.4; Carl Zeiss Microscopy, Oberkochen, Germany). Images of cultured primary neurons were retrieved with a spinning disc confocal microscope (Cell Observer SD, Carl Zeiss Microscopy, Oberkochen, Germany) equipped with a plan-apochromat 20x air objective and a plan-apochromat 63x oil immersion objective. Z-stacks were obtained from EGFP-positive neuronal somata.

For all recordings, settings including exposure time, laser intensity, and gain were kept constant across samples from different experimental groups. Each representative image is a maximum intensity projection of a z-stack.

### 2.8 Determination of GABA, TrkB, phospho-CaMKII, and cleaved caspase 3 protein expression

To evaluate the specificity of CaMKII promoter-driven transgene expression, transduced primary hippocampal neurons were stained for GABA and MAP2 to identify inhibitory neurons. The proportion of GABA-positive neurons among EGFP-positive cells was quantified by calculating the percentage of GABA/EGFP double-positive neurons relative to the total EGFP-positive population.

To assess protein expression levels of TrkB, and phosphorylated CaMKII (pCaMKII), image stacks were loaded into IMARIS 10 (Bitplane, Oxford Instruments, Abingdon, UK). Following background subtraction, three-dimensional ROIs of EGFP-positive neuronal cell somata were generated using IMARIS’ surface creation functionality. These ROIs were then used to determine the mean gray values of TrkB and pCaMKII immunoreactivity. To identify Cleaved Caspase 3 (Cl.Csp3)-positive neurons, cells were analyzed based on the immunofluorescence intensity of the somatic Cl.Csp3 signal. A threshold was set using the mean fluorescence intensity to define Cl.Csp3-positive neurons, and this threshold was consistently applied across all groups. The ratio of apoptotic cells was calculated by dividing the number of Cl.Csp3/EGFP double-positive cells by the total number of EGFP-positive neurons within each microscopic section.

### 2.9 Quantification of synaptic marker protein clusters

Quantification of synaptic marker protein clusters *in vivo* was performed as previously described (Beuter et al., 2016). Proximal dendritic segments (20 μm length) were defined as EGFP/MAP2-positive dendritic segments within 20 μm distance to the corresponding soma, while distal dendritic segments (20 μm length) were defined as those located 80 μm away (Supplementary Figure S1I). The axon initial segment of EGFP-positive neurons was defined by staining for voltage gated sodium channels (VGscs) (Supplementary Figures S1J). Pre-and postsynaptic markers were analyzed individually as described previously (Kriebel et al., 2011) To quantify synaptic marker protein clusters *in vitro*, image stacks were loaded into IMARIS 10 (Bitplane, Oxford Instruments, Abingdon, UK). Three-dimensional ROIs of the EGFP-positive neuronal cell somata were generated using IMARIS’ surface creation functionality. These ROIs were used to mask EGFP fluorescent signals and subsequently generate new image channels for the respective synaptic marker channels. Synaptic marker structures were quantified using IMARIS’ spot detection functionality, with a threshold setting maintained constant across samples from different experimental groups. The number of structures was normalized to the volume of the corresponding EGFP-based ROI to determine marker density. To determine mean cluster size of individual markers, the mean cluster volume for each neuron was analyzed after three-dimensional rendering of the respective image channels by using IMARIS’ surface creation tool. To identify colocalized pre- and postsynaptic markers *in vitro*, the IMARIS XTention MATLAB-based plugin “Spots Colocalize” was used. A maximum distance threshold of 0.7 μm between local intensity maxima was set.

### 2.10 Calcium imaging

To specifically measure calcium transients in EGFP-positive neurons, DIV14 cells were stained by replacing 50% of the neuronal medium with BrainPhys + SM1 (STEMCELL Technologies, Vancouver, Canada) containing the red-fluorescent calcium indicator 2 μM CalBryte™^*^ 590 AM (AAT Bioquest, Pleasanton, CA) Cells were incubated for 30 min at 37°C, 5% CO_2_. After exchange of culture medium, culture plates were mounted on a Cell Observer SD (Carl Zeiss Microscopy, Oberkochen, Germany). Calcium transients were recorded in imaging optimized BrainPhys + SM1 (STEMCELL Technologies, Vancouver, Canada). Recordings of 3 min of multiple cells within a microscopic field were retrieved, with two microscopic fields per well. To identify EGFP-positive neurons and enable cell specific analysis, snapshots of the CalBryte and EGFP signals were taken for each microscopic field. Somatic calcium transients were measured from individual neurons by manually drawing ROIs with Fiji software (Schindelin et al., 2012) and extracting the mean fluorescence intensity over time. A minimum of three spontaneously active EGFP-positive neurons (at least two calcium transients within a measurement) were analyzed per recording. Extracted traces were further analyzed with the Origin 2015G Peak Analyzer module (OriginLab Corporation, Northampton, MA). Traces were normalized to baseline, and peak detection threshold and minimum peak distance were kept constant across all replicates. Peak parameters [area under the curve (AUC), full width half maximum (FHWM), amplitude] were averaged across all peaks of a single neuron. Data points in the graphs are normalized to the WT mean and represent the mean of multiple cells per well.

### 2.11 Statistics

Statistical analysis was performed with GraphPad Prism 10 (GraphPad Software, Boston, MA). All data were submitted to outlier detection and tested for Gaussian distribution. The statistical tests used for assessment of significance are stated in the respective figure legends. All values are represented as mean ± S.E.M and values are stated in the respective figure legends. The *p*-values were assigned as follows: *p < 0.05; **p < 0.01; ***p < 0.001, ****p < 0.0001. Sample size is described in the respective figure legends. An independent experiment describes an individual dissection and culture of neurons. Detailed statistical values for all comparisons can be found in Supplementary Table S3.

## 3 Results

### 3.1 TrkB is required for the organization of gephyrin scaffolds *in vivo*

In a previous report, we have demonstrated a role for Bdnf–TrkB signaling for gephyrin clustering on proximal dendritic segments of primary hippocampal neurons *in vitro* (Wuchter et al., 2012). Extending this work, we prepared recombinant lentiviral vectors for the expression of miRNA targeting rat *Ntrk2* (TrkB) mRNA *in vivo*. TrkB-specific miRNAs mi162 and mi1973 were expressed under the control of a CaMKII promoter which is active in principal neurons, exclusively. Additionally, an EGFP reporter, driven by a synapsin promoter, was included to localize injections sites *in vivo*. Lentiviral vector maps and efficient knockdown of TrkB after lentiviral transduction at the mRNA and protein level has been shown recently (Beuter, 2017).

**Figure 1:**
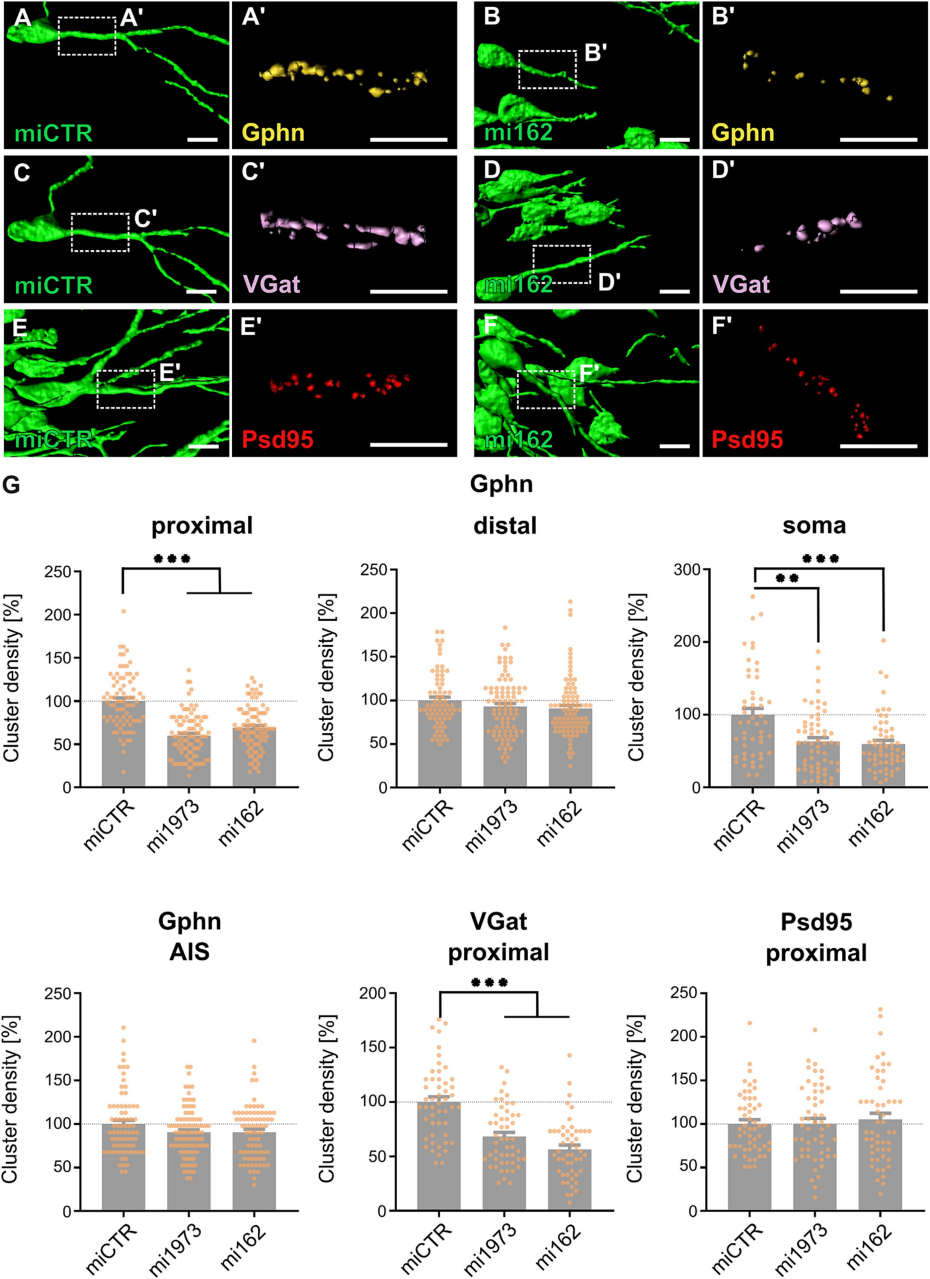
KD of TrkB reduces gephyrin cluster density specifically at perisomatic compartments of rat dentate gyrus granule neurons *in vivo*. (A–F) Representative 3D stacks of EGFP signal rendered images of miCTR and mi162 transduced granule cells. Rectangles depict enlarged areas of dendritic compartments stained for postsynaptic gephyrin (Gphn) (A’,B’), inhibitory presynaptic marker VGat (C’,D’), and the excitatory postsynaptic marker Psd95 (red, E’,F’). Scale bars: 10 μm. Correpsonding microcopic images are shown in Supplementary Figure S1C-H (G) Quantification of gephyrin cluster density on proximal and distal dendritic segments, on somata and the axon initial segment (AIS) of EGFP transduced granule cells. Numerical data are mean values ± SEM normalized to miCTR. Statistical significance was assessed by Kruskal-Wallis and one-way ANOVA with Dunn’s multiple comparison test. **p < 0.01, ***p < 0.001; non-significant comparisons are not indicated. The data shown in the histograms were obtained from three animals for each individual experimental group. Proximal dendrites: miCTR 100.0 ± 3.65; mi1973, 59.9 ± 2.60; mi162, 68.9 ± 2.63; *n* = 79–96. Distal dendrites: miCTR, 100.0 ± 3.79; mi1973, 93.0 ± 3.54; mi162, 90.8 ± 3.59; *n* = 71–90. Somata: miCTR, 100.0 ± 8.75; mi1973, 63.0 ± 5.43; mi162, 59.5 ± 5.11; *n* = 50–59. AIS: miCTR, 100.0 ± 4.16; mi1973, 90.3 ± 2.96; mi162, 90.5 ± 3.59; *n* = 75–90. VGat and Psd95 cluster densities at the proximal dendrites of DG granule cells: VGat: miCTR, 100.0 ± 4.78; mi1973, 68.1 ± 3.87; mi162, 55.5 ± 3.89; *n* = 50. Psd95: miCTR, 100.0 ± 4.82; mi1973, 100.4 ± 5.85; mi162, 105.2 ± 7.01; *n* = 52–53. For detailed statistical analysis refer to Supplementary Table S3.

Lentiviral vectors for the knockdown of TrkB (mi162 and mi1973) as well as an ineffective control (miCTR) were injected into the dorsal dentate gyrus of anaesthetized juvenile rats. Application of two independent TrkB-specific miRNAs ruled out potential off-target effects (Colon et al., 2024). After 14 days, animals were sacrificed, brains fixed and stained for synaptic marker proteins. EGFP fluorescence analysis by confocal microscopy confirmed successful transduction of granule cells of the dentate gyrus (for representative low-magnification images see Supplementary Figure S1A,B). Likewise, GABAergic synapse markers VGat (for presynapses), gephyrin (Gphn, for postsynapses) as well as a glutamatergic postsynaptic marker (Psd95) were detected on EGFP-positive somata and neurites (Figures 1A-F; see Supplementary Figure S1C–H for maximum intensity projections of confocal z-stacks). Quantification of gephyrin cluster densities revealed that TrkB knockdown by both mi162 or mi1973 significantly reduced gephyrin cluster densities on proximal dendritic segments and somata of granule cells by at least 31 and 37%, respectively, as compared to miCTR (Figure 1G). In contrast, gephyrin cluster densities on distal segments and on the axon initial segment remained unaffected. Likewise, presynaptic GABAergic marker VGat densities on proximal segments were reduced while postsynaptic glutamatergic marker Psd95 densities remained unimpaired. These findings indicate that TrkB receptor expression is required for gephyrin clustering specifically at proximal dendritic and somatic compartments of granule cells *in vivo*.

### 3.2 Different TrkB-dependent pathways modulate gephyrin clustering

For a detailed analysis of signaling pathways contributing to the TrkB-dependent regulation of gephyrin clustering, we generated a set of point mutations in the rat *Ntrk2* coding sequence for overexpression of signaling deficient TrkB mutants in dissociated primary hippocampal neurons. To this end, we mutated the rat *Ntrk2* sequence at amino acid residue 515 (Y515F) to remove the Shc binding site (further referred as TrkB SHC-), at amino acid residue 816 (Y816F) to interfere with PLCγ interaction (further referred as TrkB PLC-), and at amino acid residue 571 (K571A) to create a kinase-dead (further referred as TrkB KD) mutant. Successful mutation was confirmed by sequencing (Supplementary Figure S2A). Wild type TrkB (WT) as well as the TrkB mutants fused to an EGFP reporter downstream of a CMV promoter were expressed in NIH3T3 or HEK293 cells (Supplementary Figure S2B–D) (Minichiello et al., 2002; McCarty and Feinstein, 1998; Minichiello et al., 1998). Western Blot analysis with an antibody specific for TrkB indicated that all constructs expressed TrkB-EGFP fusion proteins at 120 kDa (Supplementary Figure S2B). A specific polyclonal TrkB antibody specific for phosphorylation of the catalytic domain showed no autophosphorylation of the TrkB KD mutant while TrkB WT, TrkB SHC- and TrkB PLC- were active in the presence or absence of Bdnf stimulation. TrkB is suggested to induce Erk1/2 activation as a downstream effector via interaction with Shc (Minichiello et al., 2002; Atwal et al., 2000). Analysis of Erk1/2 phosphorylation after overexpression of TrkB WT and TrkB mutants indicated a trend for reduced activation of Erk1 after expression of the TrkB SHC- by 54% and the TrkB KD mutant by 24% compared to TrkB WT and TrkB PLC- mutant (Supplementary Figure S2C). Moreover, PLCγ1 activation after expression of TrkB PLC- or TrkB KD mutants was reduced 73 and > 99%, respectively. While TrkB SHC- remained ineffective (Supplementary Figure S2D). In conclusion, the mutants showed the selective impairment of either TrkB activation (TrkB KD), reduced Shc-dependent signaling (TrkB SHC-) or PLCγ activation (TrkB PLC-).

Subsequently, TrkB WT and mutants were transferred into lentiviral backbones for expression under the control of a CaMKII promoter (Supplementary Figure S3A). All mutants were expressed in primary hippocampal neurons as detected by EGFP fluorescence (Supplementary Figures S3B–E). To evaluate cell specificity of the CaMKII promoter, we quantified the percentage of GABA-positive neurons among EGFP-transduced neurons. A total 10.96 ± 2.04% of all EGFP-positive cells expressed GABA indicating no specificity for excitatory neurons *in vitro* (Supplementary Figures S3F–G). Quantitative immunofluorescence analysis with an antibody specific for TrkB revealed an equal increase in TrkB expression in the presence of all constructs in comparison to neurons transduced with an EGFP expressing control vector (Supplementary Figures S4A–F). To rule out that overexpression of TrkB SHC- reduces neuronal survival due to impaired Akt signaling (Atwal et al., 2000), the percentage of caspase 3-positive cells in the population of EGFP-transduced neurons was determined. Quantification revealed no impact on the viability of hippocampal neurons by any TrkB mutant (Supplementary Figures S4G–H).

**Figure 2:**
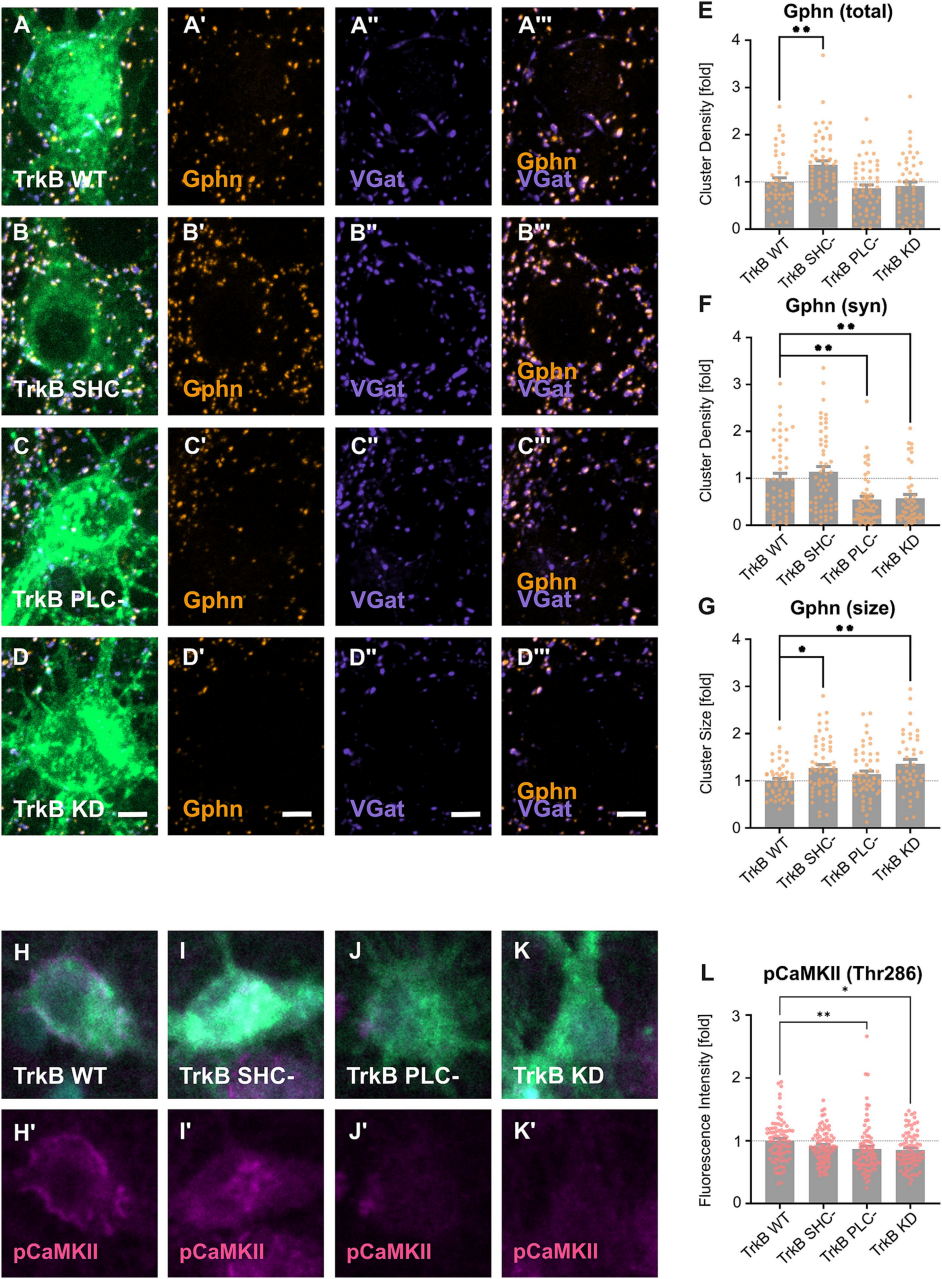
Differential contributions of TrkB signaling pathways to the regulation of gephyrin clustering *in vitro*. (A–D) Representative images of primary hippocampal neurons transduced with EGFP-tagged TrkB WT (A) and mutants (B–D) and stained for the inhibitory pre- and postsynaptic markers gephyrin (Gphn, A’,B’,C’,D’), VGat (A,”B”,C”,D”), and Gphn/VGat (A”’,B”’,C”’,D”)’. Scale bars: 10 μm (A–D) and 2.5 μm (A’–A”’, B’-B”’, C’–C”’, D’–D”’). Overview images are shown in Supplementary Figure S5. (E) Quantification of total gephyrin cluster densities on EGFP-positive somata: TrkB WT, 1.00 ± 0.08; TrkB SHC-, 1.36 ± 0.09; TrkB PLC-, 0.87 ± 0.07; TrkB KD, 0.91 ± 0.09; *n* = 45–55 somata from 4 independent experiments. (F) Quantification of synaptic gephyrin cluster densities of EGFP positive somata (TrkB WT, 1.00 ± 0.11; TrkB SHC-, 1.14 ± 0.12; TrkB PLC-, 0.54 ± 0.07; TrkB KD, 0.57 ± 0.08; *n* = 44–54 somata from 4 independent experiments). (G) Quantification of total mean gephyrin cluster size of EGFP positive somata: TrkB WT, 1.00 ± 0.05; TrkB SHC-, 1.27 ± 0.07; TrkB PLC-, 1.14 ± 0.07; TrkB KD = 1.36 ± 0.10; *n* = 43–60 somata from 4 independent experiments. (H–K) Representative images of primary hippocampal neurons transduced with TrkB-EGFP WT (H) and mutants (I–K) and stained for phosphorylated CaMKII (pCaMKII, H’,I’,J’,K’) and pan CaMKII (CaMKII, H”, I”, J,” K”). Scale bar: 2.5 μm. Overview images are shown in Supplementary Figure S7. (L) Quantification of the mean fluorescence intensity of labeled pCaMKII in EGFP positive somata: TrkB WT, 1.00 ± 0.04; TrkB SHC-, 0.91 ± 0.03; TrkB PLC- = 0.87 ± 0.05; TrkB KD, 0.85 ± 0.03; *n* = 80–81 somata from three independent experiments. (M) Quantification of the mean fluorescence intensity of labeled CaMKII in EGFP positive somata (TrkB WT, 1.00 ± 0.05; TrkB SHC-, 0.95 ± 0.04; TrkB PLC- = 0.94 ± 0.06; TrkB KD, 0.83 ± 0.04; *n* = 79–80 somata from three independent experiments). Numerical data are means ± SEM; Values are normalized to WT. Statistical significance was assessed by Kruskal-Wallis with Dunn’s multiple comparison test (*p < 0.05, **p < 0.01; non-significant comparisons are not noted). For detailed statistical analysis refer to Supplementary Table S3.

Based on our observation that TrkB is required for gephyrin clustering on cell bodies *in vivo*, we analyzed gephyrin clustering on somata after transduction of TrkB WT as well as TrkB mutants in hippocampal neurons *in vitro* (Figure 2). EGFP-positive transduced neurons expressing TrkB WT (Figure 2A), TrkB SHC- (Figure 2B), TrkB PLC- (Figure 2C) or TrkB KD (Figure 2D) were stained for gephyrin (Gphn) and VGat. In particular, we determined gephyrin cluster densities across the whole somatic compartment (total, Figure 2E) and of the subpopulation of synaptic gephyrin clusters defined by the apposition to presynaptic VGat punctae (syn, Figure 2F). Additionally, gephyrin cluster size was measured on whole neuronal somata (Figure 2G). Overview images are given in Supplementary Figures S5A–D. Analysis of total gephyrin cluster density revealed an increase after expression of the TrkB SHC- mutant, while TrkB PLC- or TrkB KD overexpression remained ineffective (Figure 2E). Focusing on synaptic gephyrin cluster density, TrkB PLC- and TrkB KD reduced synaptic gephyrin density while the TrkB SHC- mutant was ineffective (Figure 2F). Hence, TrkB activity and TrkB-dependent activation of PLCγ is required for synaptic gephyrin clustering. Examination of gephyrin cluster size revealed an increase after expression of TrkB SHC- and TrkB KD (Figure 2G). We conclude that TrkB-dependent Shc signaling is involved in the negative control of gephyrin cluster size. Gephyrin is required for the localization of GABA_A_ receptors to postsynaptic sites (Kneussel et al., 1999). Expression of glutamatergic marker Psd95, both total and synaptic remained unaffected after overexpression of TrkB WT and mutants, indicating selectivity for gephyrin clustering (Supplementary Figures S6A–D). In conclusion, the results imply a dual role of TrkB induced signaling to gephyrin regulation. Synaptic gephyrin clustering is positively regulated by TrkB-PLCγ signaling while gephyrin cluster size is negatively regulated by TrkB-Shc-dependent mechanisms.

Calcium signaling was previously shown to induce gephyrin clustering through calmodulin-dependent protein kinase II (CaMKII) (Petrini et al., 2014; Flores et al., 2015). Since Bdnf and TrkB-PLCγ signaling are known to activate CaMKII (Minichiello et al., 2002; Zeng et al., 2010), we asked whether the TrkB mutants alter the activation of CaMKII (Zeng et al., 2010). CaMKII autophosphorylation and activation was analyzed by quantitative immunofluorescence analysis with an antibody specific for phosphoThr286 (Figures 2H–K). Overexpression of TrkB PLC- and TrkB KD significantly reduced CaMKII phosphorylation indicating that TrkB activity is indeed required for the activation of CamKII (Figure 2L; for overview see Supplementary Figure S7A–D). Overexpression of the TrkB SHC- - did not impair CaMKII phosphorylation, significantly. Thus, loss of synaptic gephyrin clusters after TrkB PLC- and KD overexpression is accompanied by reduced CaMKII activity.

### 3.3 TrkB-dependent PLCγ signaling modulates neuronal activity

**Figure 3:**
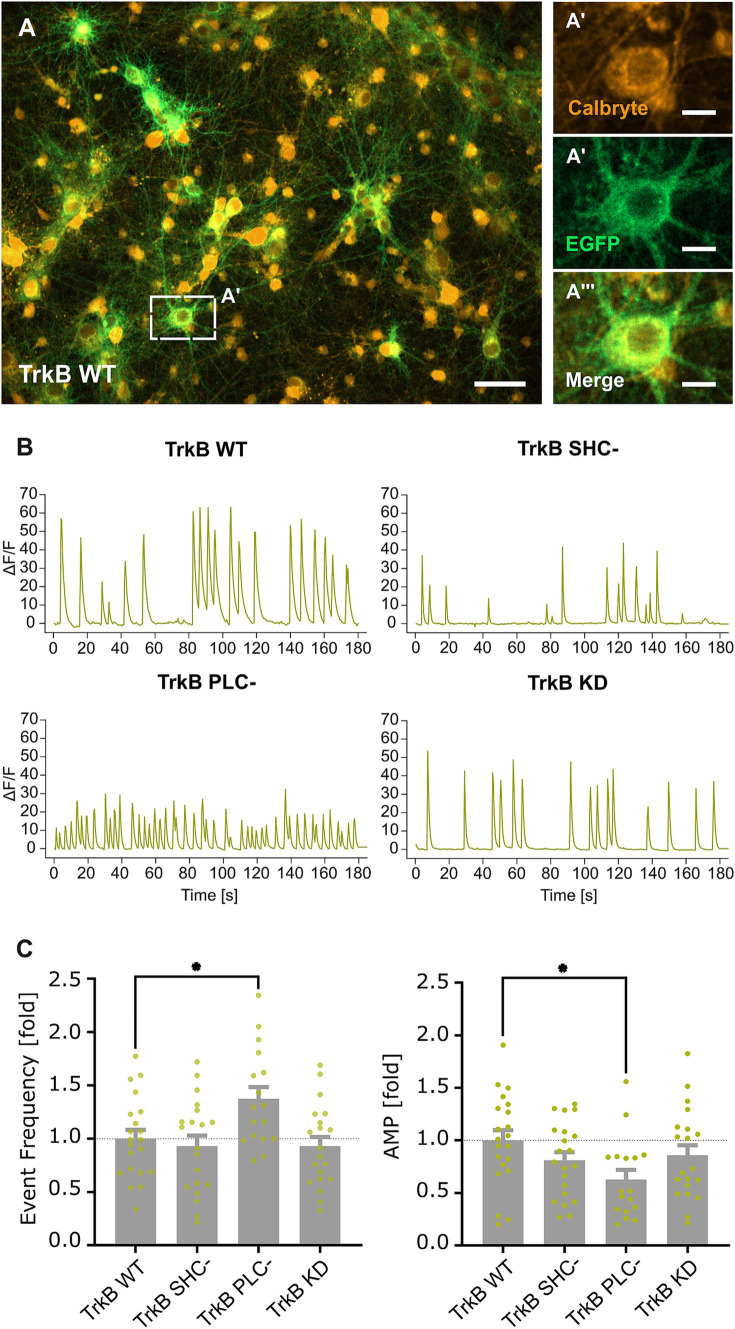
TrkB-PLCγ dependent reduction in inhibitory synapses increases excitability of primary neurons *in vitro*. (A) Representative image of TrkB WT transduced hippocampal primary neurons stained with Calbryte 590 AM (A’), EGFP (A”), EGFP/ Calbryte 590 AM (A”’). The dashed rectangle indicates the enlarged section depicted in A’–A”’. Scale bar: 10 μm (A) and 2.5 μm (A’–A”’). (B) Example calcium signal traces recorded from somata of EGFP-positive neurons. (C) Quantification of mean event frequencies and amplitudes (AMP) of calcium transients in neurons expressing TrkB WT and mutants. AMP: TrkB WT, 1.00 ± 0.10; TrkB SHC-, 0.81 ± 0.08; TrkB PLC-, 0.63 ± 0.09; TrkB KD, 0.86 ± 0.10; Event Frequency: TrkB WT, 1.00 ± 0.08; TrkB SHC-, 0.93 ± 0.10; TrkB PLC-, 1.37 ± 0.11; TrkB KD, 0.93 ± 0.09; *n* = 17–21 wells from 7 independent experiments. Numerical data are values ± SEM normalized to WT. Statistical significance was assessed by one-way ANOVA with Dunn’s multiple comparison test (**p < 0.01; non-significant comparisons are not noted). For detailed statistical analysis refer to Supplementary Table S3.

Impaired synaptic gephyrin and concomitant GABA_A_ receptor clustering may result in a change in neuronal activity of glutamatergic neurons. TrkB WT and TrkB mutants expressed under the control of the CaMKII promoter were transduced into hippocampal neurons. Principal neurons were identified through EGFP fluorescence (Figure 3A) and subsequently submitted to calcium imaging for measuring neuronal activity (Figure 3B). In accordance with reduced inhibitory synapse formation (Figure 2F), overexpression of TrkB PLC- significantly increased the frequency of calcium transients, while the amplitude was decreased (Figure 3C). However, no changes of calcium transients were observed after overexpression of the TrkB SHC- mutant. Eventually, opposing effects of SHC signaling reducing gephyrin cluster size (Figure 2G) and PLCγ signaling inducing synaptic gephyrin clustering (Figure 2F) mask each other after the combined inhibition of both pathways with the TrkB KD mutant.

### 3.4 Chem iLTP induces TrkB-dependent gephyrin clustering

**Figure 4:**
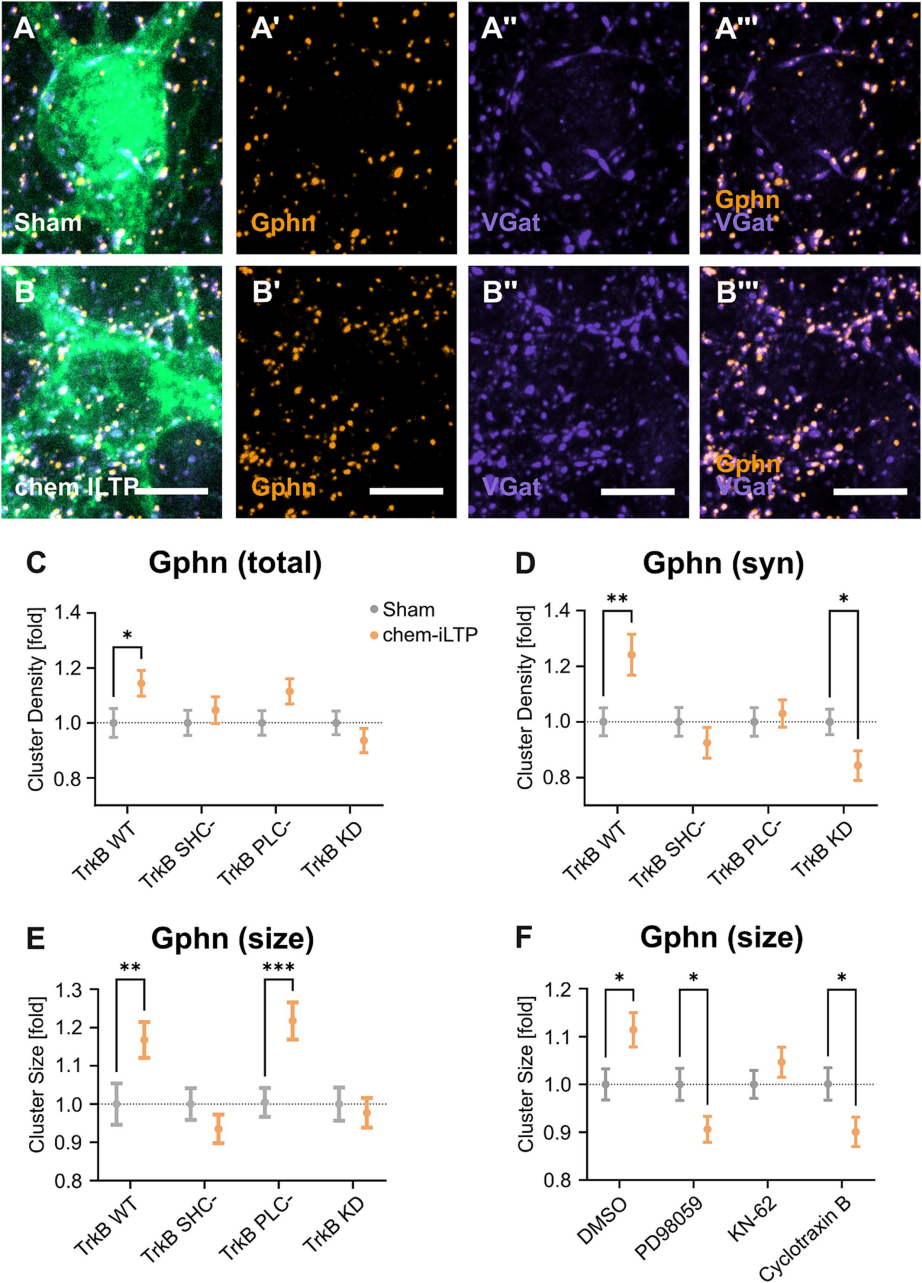
Chemically induced long-term potentiation (chem iLTP) at inhibitory synapses relies on TrkB signaling. (A, B) Representative images of TrkB WT expressing primary neurons after treatment with sham solution (A) or NMDA+CNQX (B) for chem iLTP induction. (A’,B’) Gphn staining. (A”,B”) VGat staining. (A”’,B”’) Gphn/VGat. Scale bars: 5 μm. Overview images and individual datapoints are shown in Supplementary Figure S8. (C–G) Analysis of gephyrin clustering in neurons expressing TrkB WT and mutants after chem-iLTP or sham treatment as indicated. Gray dots: sham treatment. Orange dots: chem iLTP. Numerical data are means ± SEM, obtained from three independent experiments.Values are normalized to the corresponding untreated group. Statistical significance was assessed by two-way ANOVA with Tukey’s multiple comparison test (*p < 0.05, **p < 0.01, ***p < 0.001; non-significant comparisons are not noted). For detailed statistical values refer to Supplementary Table S3. (C) Total somatic gephyrin cluster densities: TrkB WT sham, 1.00 ± 0.05; TrkB WT chem iLTP, 1.14 ± 0.05; TrkB SHC- sham, 1.00 ± 0.05; TrkB SHC- chem iLTP, 1.05 ± 0.05; TrkB PLC- sham, 1.00 ± 0.04; TrkB PLC- sham chem iLTP, 1.12 ± 0.05; TrkB KD, 1.00 ± 0.04; TrkB KD chem iLTP, 1.05 ± 0.05; *n* = 80–90 somata. (D) Synaptic somatic gephyrin cluster densities. TrkB WT sham, 1.00 ± 0.05; TrkB WT chem iLTP, 1.24 ± 0.07; TrkB SHC- sham, 1.00 ± 0.05; TrkB SHC- chem iLTP, 0.93 ± 0.06; TrkB PLC- sham, 1.00 ± 0.05; TrkB PLC- chem iLTP, 1.03 ± 0.05; TrkB KD, 1.00 ± 0.05; TrkB KD chem iLTP, 0.84 ± 0.05; *n* = 80–90 somata. (E) Mean gephyrin cluster size. TrkB WT sham, 1.00 ± 0.05; TrkB WT chem iLTP, 1.17 ± 0.05; TrkB SHC- sham, 1.00 ± 0.04; TrkB SHC chem iLTP, 0.94 ± 0.04; TrkB PLC- sham, 1.00 ± 0.04, TrkB PLC- chem iLTP, 1.22 ± 0.05; TrkB KD, 1.00 ± 0.04; TrkB KD chem iLTP, 0.98 ± 0.04; *n* = 78–90 somata. (F) Mean gephyrin cluster size. CTR sham, 1.00 ± 0.03; CTR chem iLTP, 1.11 ± 0.04; PD98059 sham, 1.00 ± 0.03; PD98059 chem iLTP, 0.90 ± 0.03; KN-62 sham, 1.00 ± 0.03; KN-62 chem iLTP, 1.05 ± 0.03; Cyclotraxin B sham, 1.00 ± 0.03; Cyclotraxin B chem iLTP, 0.90 ± 0.03; *n* = 82–89 somata. Corresponding microscopic images are shown in Supplementary Figure S9.

Chemically induced inhibitory long-term potentiation (chem iLTP) utilizes a moderate calcium elevation protocol with the application of NMDA and CNQX. This method induces gephyrin clustering dependent on CaMKII activity, to simulate a homeostatic response (Petrini et al., 2014). To further elucidate a role for TrkB to chem iLTP-induced gephyrin clustering, we transduced expression vectors for TrkB WT and its mutants into primary hippocampal neurons and asked for an impact of TrkB on chem iLTP-induced gephyrin clustering. In the presence of TrkB WT, chem iLTP increased synaptic gephyrin clustering in our experimental system (Figures 4A,B). Overexpression of TrkB KD as well as of TrkB SHC- and TrkB PLC- reduced total and synaptic gephyrin clustering to base line levels, suggesting that TrkB activity as well as activation of both signaling pathways are a requirement for chem iLTP-induced gephyrin clustering (Figures 4C,D). The findings suggest Shc signaling to induce gephyrin clustering in chem iLTP while it reduces gephyrin clustering under unstimulated basal conditions (Figures 2E,G). In contrast, chem iLTP-induced increase in gephyrin cluster size was not affected by TrkB PLC- while this was completely abolished by TrkB SHC- and TrkB KD (Figure 4E). Therefore, chem iLTP-induced gephyrin cluster expansion is mainly triggered by TrkB-dependent Shc signaling. For further confirmation, we added selective antagonists for Mek1 (PD98059), CaMKII (KN-62), and TrkB (Cyclotraxin B) to TrkB WT overexpressing neurons to interfere with TrkB activity, Shc- and PLC-dependent signaling with an independent approach (Figure 4F). The results show that total gephyrin cluster size is reduced after application of each of the three compounds. Compared to TrkB PLC- overexpression (Figure 4E) a pronounced inhibition was observed by the CaMKII inhibitor KN-62. The results may be explained by additional mechanisms of chem iLTP-induced calcium increase, such as the calcium influx through NMDA receptors (Petrini et al., 2014).

## 4 Discussion

**Figure 5:**
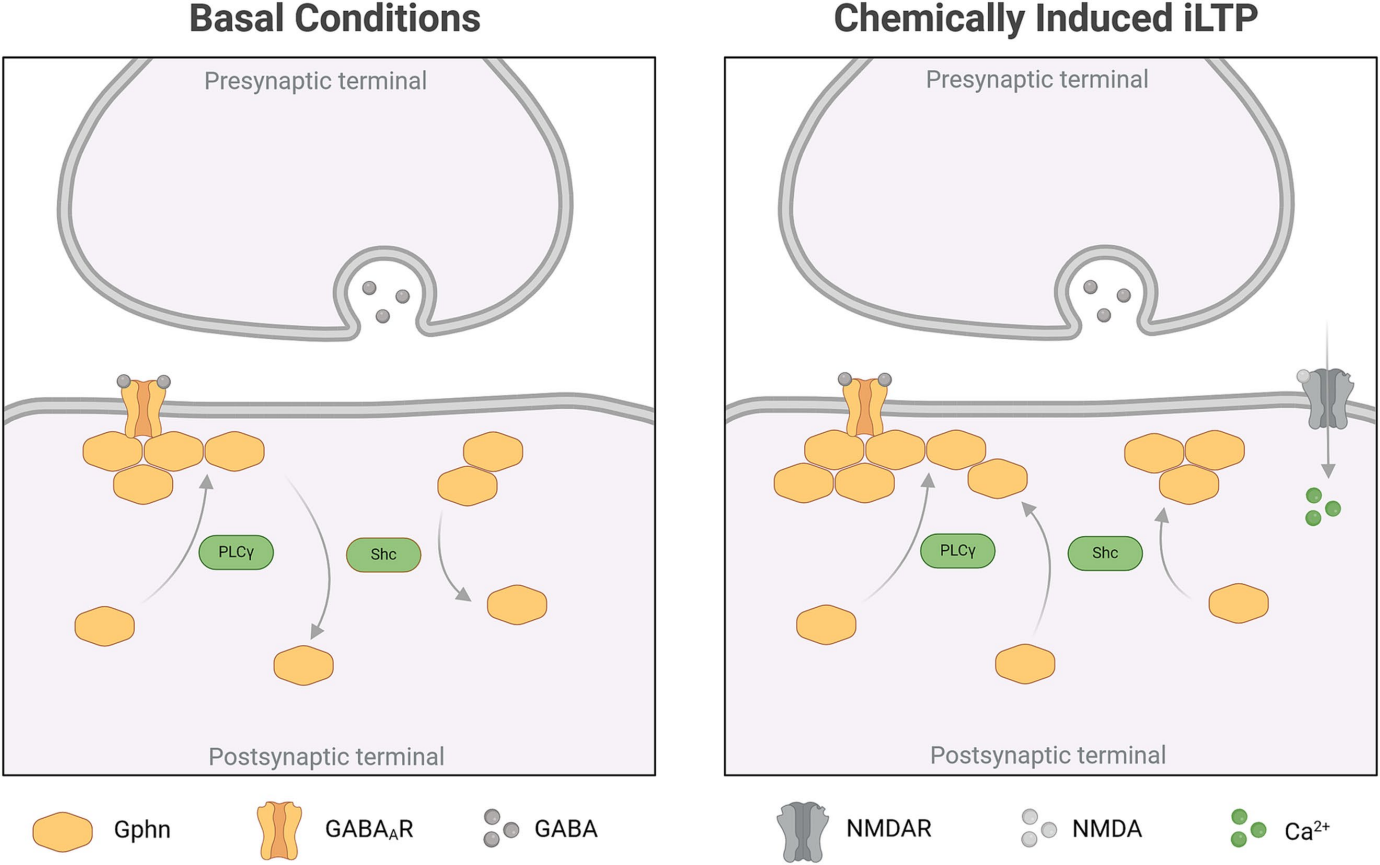
Summary of TrkB-dependent gephyrin clustering under basal conditions and after chem iLTP (Created in BioRender.com).

Here, we show, that TrkB expression is required for gephyrin clustering at neuronal somata and on proximal dendrites. Subsequent, comprehensive analysis of gephyrin clustering on somata after overexpression of TrkB mutants *in vitro* revealed that TrkB-PLCγ signaling is required for gephyrin clustering at synaptic sites while TrkB-Shc-dependent signaling has a dual role in decreasing gephyrin cluster size under unstimulated conditions while increasing gephyrin cluster size after chem iLTP (for overview see Figure 5 and Table 1).

**Table 1:** Overview of TrkB-dependent gephyrin clustering: key observations.

Basal conditions			
Observation	TrkB SHC-	TrkB PLC-	TrkB KD
Gphn cluster size	🠩	=	🠩
Gphn cluster density	🠩	=	=
Gphn syn. Cluster density	=	🠫	🠫
E/I balance	=	🠩 Excitability	🠩 Excitability
Calcium Freq	=	🠩	=
pCaMKII	=	🠫	🠫

chem iLTP				
Observation	TrkB WT	TrkB SHC-	TrkB PLC-	TrkB KD
Gphn cluster size	🠩	=	=	=
Gphn cluster density	🠩	=	=	=
Gphn syn. Cluster density	🠩	=	🠩	=

Previous reports have demonstrated that interference with gephyrin clustering affects the frequency and amplitude of miniature inhibitory postsynaptic currents (mIPSCs) (Pizzarelli et al., 2020). Specifically, an increase in the size of gephyrin clusters correlates with a higher amplitude of GABAergic mIPSCs, suggesting that larger clusters enhance inhibitory signaling (Gonzalez-Forero et al., 2005). Conversely, disruption of gephyrin clusters leads to a decrease in both the amplitude and frequency of spontaneous GABAergic synaptic currents, indicating a weakening of inhibitory function (Yu et al., 2007). Although mIPSCs were not directly measured in the current study, the observed increase in neuronal excitability in calcium imaging, associated with decreased synaptic gephyrin cluster density following TrkB-PLC- overexpression implies a significant impact on inhibitory activity. Our findings suggest that TrkB controls gephyrin clustering through PLCγ signaling. The contrasting outcomes observed with the kinase-dead TrkB mutant could be possibly explained by the observed increase in gephyrin cluster size, which, despite a reduction in synaptic gephyrin cluster density, might compensate for the diminished inhibitory input by strengthening GABAergic synapses. Further investigations, including the measurement of mIPSCs following TrkB mutant overexpression, could clarify the functional consequences of TrkB-dependent regulation of gephyrin on GABAergic synapses.

Bdnf and TrkB are abundantly expressed in hippocampal granule cells, both in excitatory as well as in inhibitory synapses (Drake et al., 1999) playing a crucial role in synaptic function, LTP and hippocampal memory (Minichiello, 2009; Sun et al., 2023). The hippocampus harbors various types of interneurons to be discriminated by the specific target compartment addressed on principal neurons (Pelkey et al., 2017; Freund and Buzsaki, 1996). Previous studies imply that NPAS4/Bdnf/TrkB signaling is essential for the stabilization of inhibitory synapses, particularly at the somata of principal neurons of CA1 pyramidal neurons of the hippocampus (Bloodgood et al., 2013). Accordingly, our findings confirm TrkB to govern gephyrin clustering at somata of granule cells in the DG while an impact on interneuron terminals on proximal dendrites has not been reported so far. Besides TrkB, further receptor tyrosine kinases including hepatocyte growth factor receptor Met (Jeckel et al., 2021) and ephrin receptor EphA7 (Beuter et al., 2016) have been shown to regulate GABAergic synapse stability in the DG while it is unknown whether Met controls compartment-specific gephyrin clustering. Interestingly, EphA7 shares with TrkB the same target compartment on somata. However, EphA7 expression is required for the stabilization of parvalbumin-positive basket cell terminals on granule cells (Beuter et al., 2016). In contrast, NPAS4-induced Bdnf/TrkB signaling accounts for the regulation of cholecystokinin (CCK)-expressing basket cells on CA1 pyramidal neurons (Hartzell et al., 2018). Therefore, both receptors may address the formation or stabilization of different types of basket cell input which is of functional importance given that parvalbumin-positive basket cells belong to the class of fast spiking interneurons while CCK-positive cells are regular spiking interneurons (Savanthrapadian et al., 2014).

Past reports have described opposing findings regarding the action of Bdnf and the function of signaling pathways contributing to gephyrin clustering. Based on previous observations that TrkB signaling is involved in gephyrin clustering, we have here undertaken an attempt to reconcile these contradictory findings by the overexpression of TrkB point mutants which selectively impair cognate TrkB signaling pathways. Bdnf has been reported to act differently on gephyrin clustering depending on the temporal control of Bdnf application. Short term application reduced gephyrin clustering, while long term application increased gephyrin expression (Wuchter et al., 2012; Mou et al., 2013). In principle, this was a first hint that Bdnf–TrkB signaling may exert opposing effects on gephyrin clustering. In this study, long term expression of TrkB variants simulated chronic alterations, which resulted in a reduction of inhibitory synapses either through *in vivo* TrkB knockdown or the overexpression of kinase-dead and PLC- TrkB mutants, while overexpression of the TrkB-SHC- mutant in dissociated neurons led to an increase in gephyrin expression. These findings suggest that the opposing effects observed with different Bdnf stimulations are driven by distinct mechanisms within the TrkB signaling pathways. Further studies are needed to evaluate the function of these mutants in the context of acute Bdnf stimulation. Another report unambiguously showed that gephyrin phosphorylation through Erk or GSK3β induced calpain-dependent degradation of gephyrin, while our results suggest that Erk and PI3K-Akt–mTOR signaling, both triggered by Shc, are required for the gephyrin clustering (Tyagarajan et al., 2013; Wuchter et al., 2012). Increased gephyrin clustering in the presence of the overexpressed TrkB SHC- mutant now implies that removal of gephyrin becomes indeed facilitated by the corresponding signaling pathways. In contrast, we observed a loss in the ability to increase synaptic gephyrin cluster density and size following chem iLTP in the presence of TrkB SHC- expression. The findings suggest opposing functions of TrkB-Shc-dependent signaling either for gephyrin degradation under basal conditions versus activity-dependent induction of synaptic gephyrin clustering.

Chem iLTP has been shown to account for homeostatic control of gephyrin clustering dependent on CaMKII-activation (Petrini et al., 2014; Flores et al., 2015). The initial findings were explained by increased calcium influx through NMDA receptors which induce CaMKII accumulation at inhibitory synapses for subsequent phosphorylation of gephyrin and GABA_A_Rs. Gephyrin is phosphorylated at S305 by CaMKII, which is sufficient and necessary for inhibitory synapse formation in response to neuronal activity (Flores et al., 2015). However, the authors also considered additional mechanisms that can result in a moderate intracellular calcium concentration and activation of CaMKII needed for iLTP, such as the release of calcium from internal stores (Minichiello, 2009). In this line, we have shown that TrkB activity is required for chem iLTP-induced gephyrin clustering. TrkB is widely known for its role in regulating LTP at synaptic spines (Minichiello, 2009; Johnstone and Mobley, 2020). Our observations imply an additional role for TrkB in mediating homeostatic effects by regulating the potentiation of inhibitory synapses via gephyrin clustering. Under basal conditions, activation of CamKII becomes reduced upon overexpression of the TrkB PLC- and KD mutants. This is consistent with a previous study providing evidence for TrkB-PLCγ signaling being required for proper inhibitory synapse function in rodents (Yang et al., 2017). Several lines of evidence point at a contribution of Bdnf–TrkB signaling for chem iLTP-induced gephyrin clustering through calcium release from internal stores and activation of CaMKII.

## Data Availability

The original contributions presented in the study are included in the article/Supplementary material, further inquiries can be directed to the corresponding author.

## Footnotes

^1^ http://n2t.net/addgene:32500
