# Supplementary material for: Dissection of signaling pathways regulating TrkB-dependent gephyrin clustering
Source: Front Mol Neurosci. 2024 Oct 24;17:1480820. doi: 10.3389/fnmol.2024.1480820 (PMC11556255; doi:10.3389/fnmol.2024.1480820)
Supplement: Supplementary file 1 [file Data_Sheet_1.docx]

Supplementary Material


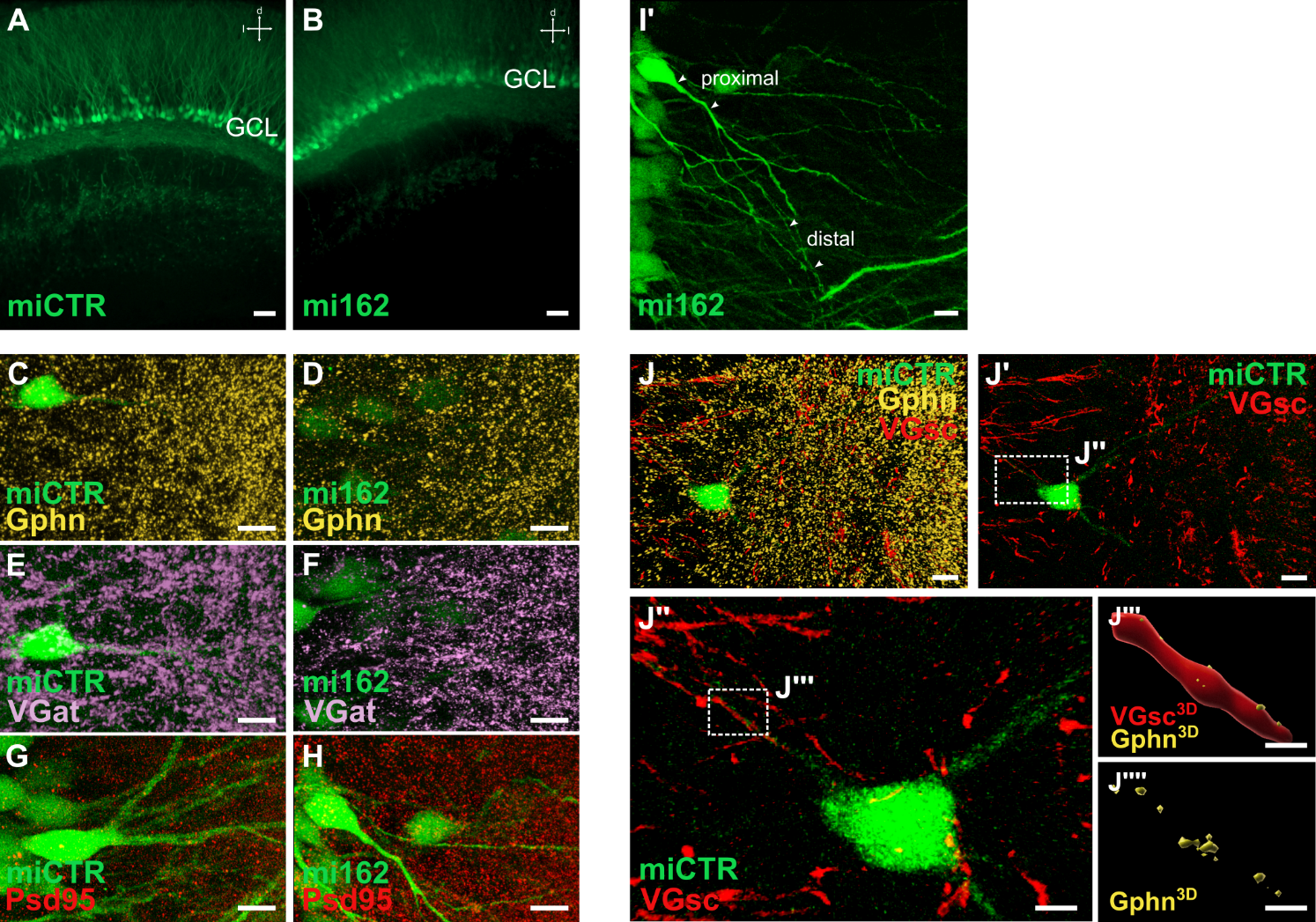


**Figure S1: Representative images of miCTR and mi162 (miTrkB) transduced DG granule cells stained for pre- and postsynaptic markers. (A-B)** Representative images of miCTR and mi162 (miTrkB) transduced cells in the granule cell layer (GCL) of the rat dentate gyrus. The cross indicates the orientation of the displayed slice (d = dorsal, l = lateral). **(C-H)** Representative maximum intensity projections (MIP) of transduced granule neurons stained for Gphn (C, D), VGat (E, F) and Psd95 (G, H). **(I)** Representative MIP of transduced granule neurons. White arrowheads indicate the 20 µm dendritic sections defined as proximal (within 20 µm from soma) and distal (80 µm distance from soma). **(J)** Representative MIP of transduced granule neurons stained for Gphn and VGsc. J’ highlights the VGsc staining in the dentate gyrus, whereas the dashed rectangle indicates the inset enlarged in J’’. J’’ represents the 3D rendered EGFP-positive neuron and the corresponding VGsc-positive AIS, which was used in J’’’ to detect Gphn clusters specifically located at the AIS (J’’’’). Scale Bars: 50 µm (A, B), 10 µm (C-H, I, J, J’), 5 µm (J’’), 3 µm (J’’’, J’’’’).


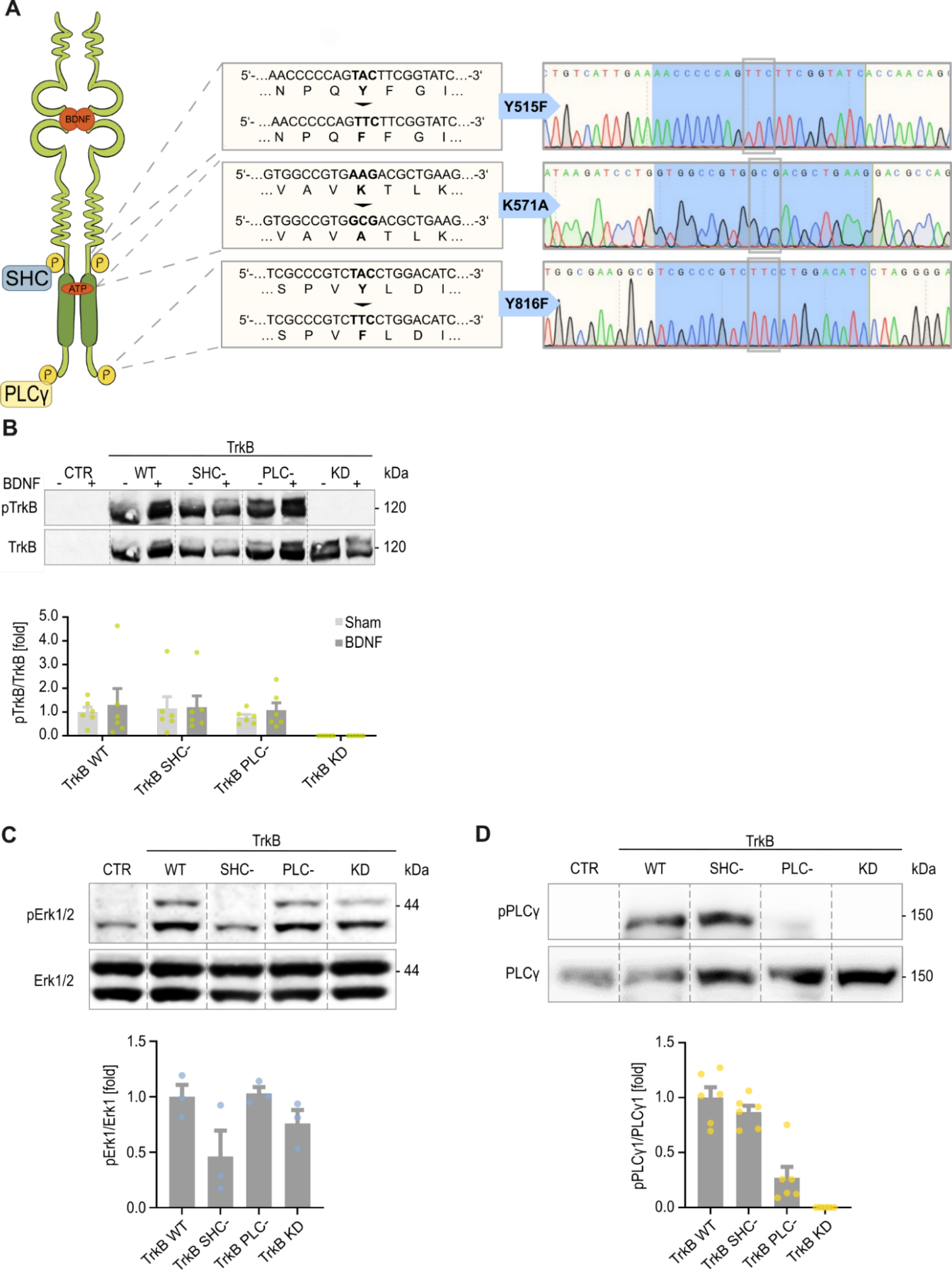


**Figure S2: Mutation of rat TrkB cds results in TrkB mutants deficient in the induction of specific signaling pathways**. (**A)** Schematic representation of the site-directed mutagenesis of rat *Ntrk2* (TrkB) open reading frame (middle panel) for the generation of TrkB mutants Y515F, K571A and Y816F. Successful mutation was assessed by Sanger sequencing. (**B)** Representative immunoblot of HEK293 cells transfected with the TrkB WT and mutants and the corresponding quantification of the ratio of phosphorylated to total TrkB protein. TrkB WT sham, 1 ± 0.21; TrkB WT Bdnf, 1.30 ± 0.69; TrkB SHC- sham, 1.15 ± 0.50; TrkB SHC- Bdnf, 1.21 ± 0.47; TrkB PLC- sham, 0.78 ± 0.12; TrkB PLC- Bdnf, 1.08 ± 0.31; TrkB KD sham, 0.002 ± 0.31; TrkB KD Bdnf, 0.001 ± 0.0006; n = 6 independent experiments. (**C)** Representative immunoblot of NIH3T3 cells transfected with the TrkB WT and mutants and the corresponding quantification of the ratio of phosphorylated to total Erk1 protein. TrkB WT, 1 ± 0.11; TrkB SHC-, 0.46 ± 0.23; TrkB PLC-, 1.03 ± 0.06; TrkB KD, 0.76 ± 0.12; n = 3 independent experiments. (**D)** Representative immunoblot of HEK293 cells transfected with the TrkB WT and mutants and the corresponding quantification of the ratio of phosphorylated to total PLCγ1 protein. TrkB WT, 1 ± 0.09; TrkB SHC-, 0.87 ± 0.06; TrkB PLC-, 0.27 ± 0.10; TrkB KD, 0.001 ± 0.0005; n = 6 independent experiments.


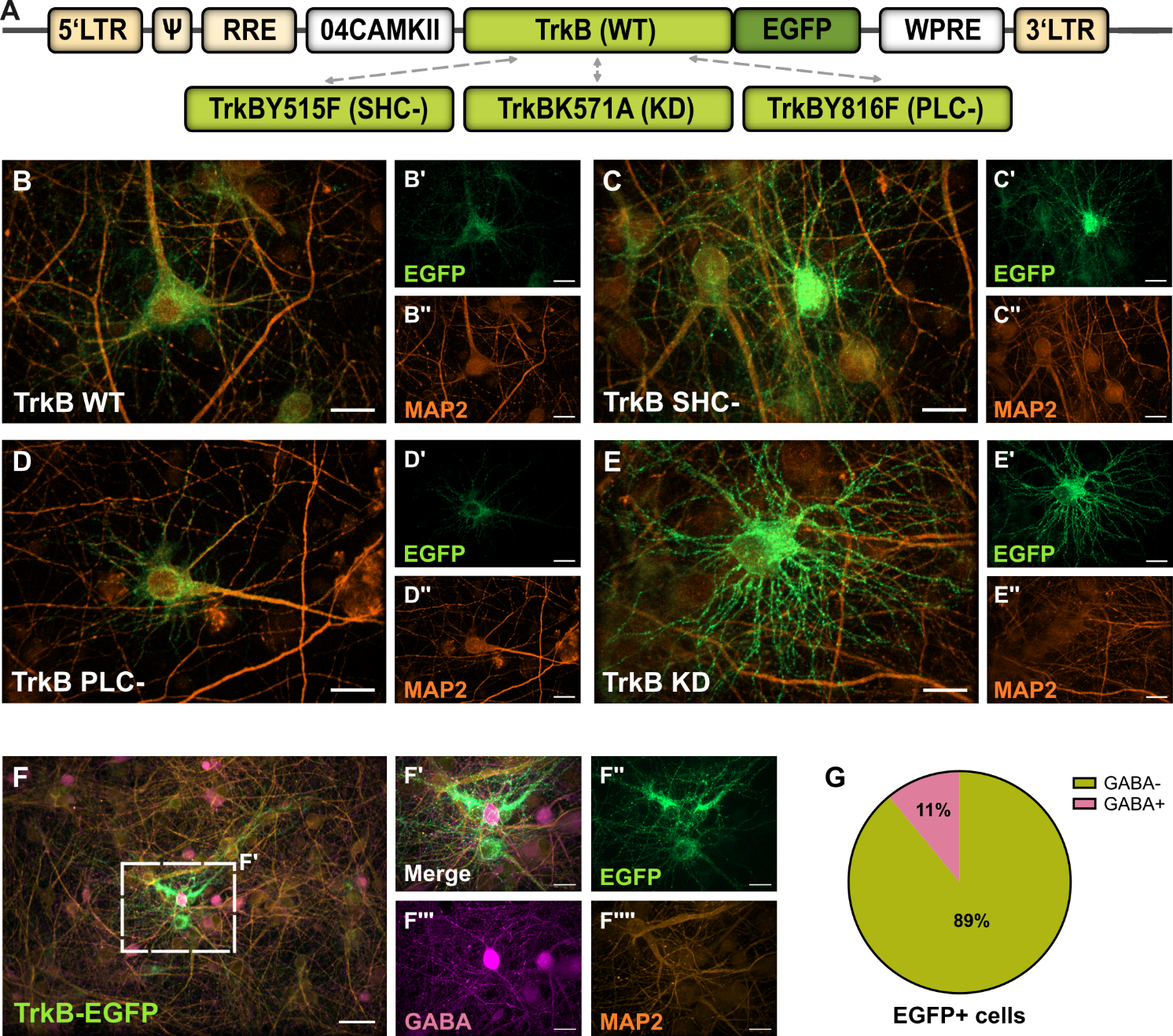


**Figure S3: Primary hippocampal neurons express the EGFP-tagged TrkB WT and primarily transduce excitatory neurons.** (**A)** Schematic representation of the lentiviral vector harboring a shortened CAMKII promotor, the TrkB-EGFP transgene and a WPRE sequence. (**B-E)** Representative images of primary hippocampal neurons 11 days post transduction of TrkB-EGFP WT (B) and mutants (C-E) stained with MAP2. EGFP signals are shown in B’, C’, D’, E’, MAP2 staining in B’’, C’’, D’’, E’’. Scale bars: 10 µm. (**F)** Representative images of primary hippocampal neurons transduced with the lentiviral vector harboring the TrkB-EGFP transgene, driven under the shortened CAMKII promotor and stained for GABA and MAP2. The dashed rectangles indicate the enlarged sections depicted in F’, F’’, F’’’, F’’’’. Scale Bars: 50 µm (F) and 10 µm (F’-F’’’’). (**G)** Quantification of GABA positive transduced primary neurons (GABA+, 10.96 % ± 2.04 %; GABA-, 89.04 % ± 2.04 %, n = 36 microscopic sections obtained from three independent experiments).


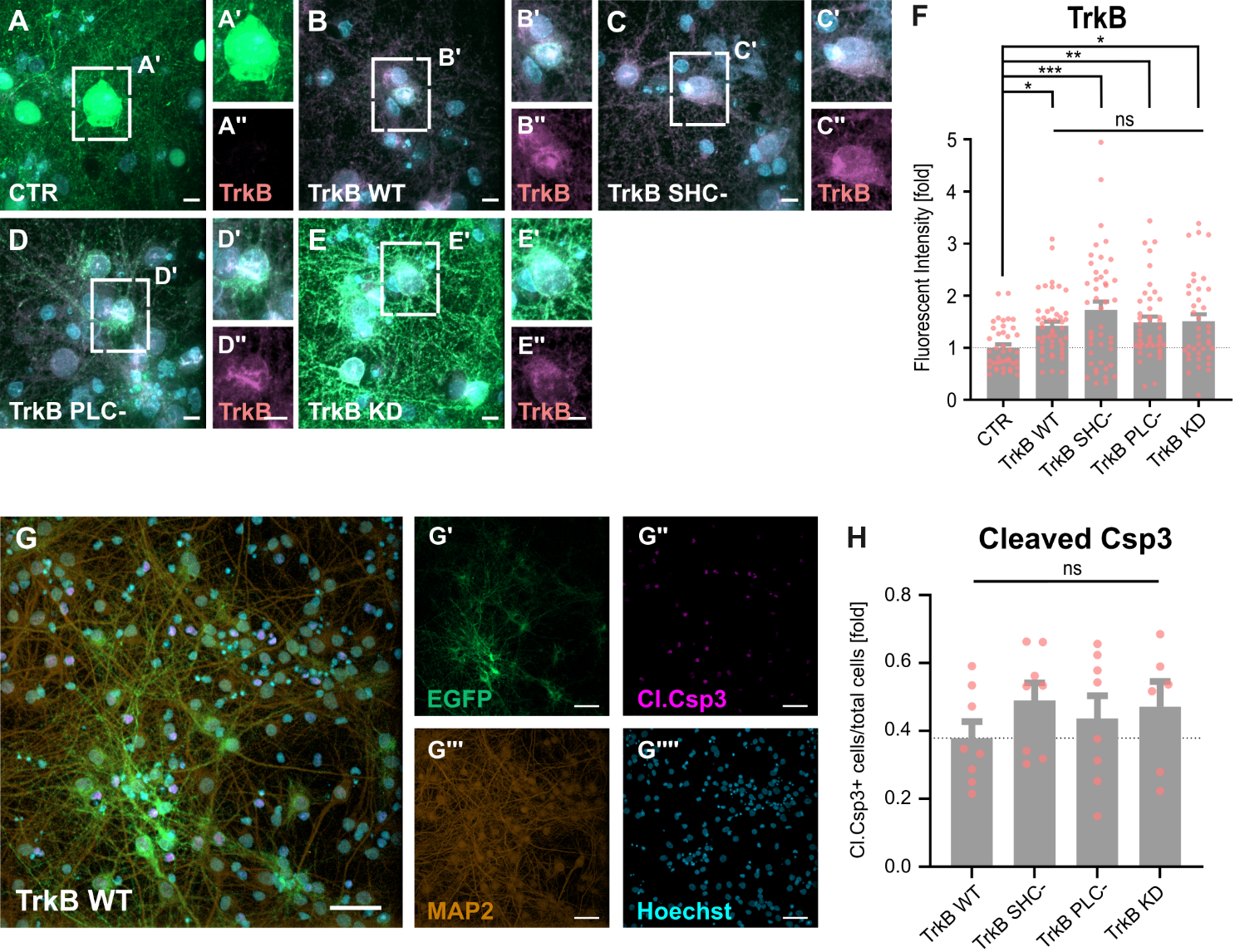


**Figure S4: Lentiviral transduction of primary hippocampal neurons with TrkB WT and mutants results in TrkB overexpression and TrkB mutants and do not alter induced cell death** (**A-E)** Representative images of primary hippocampal neurons transduced with either a lentiviral control vector containing only EGFP (CTR, A) or the TrkB WT (B) or mutants (C, D, E), stained for TrkB and Hoechst. The dashed rectangles indicate the enlarged sections depicted in A’, B’, C’, D’ and E’. TrkB signals are shown in A’’, B’’, C’’, D’’, E’’. Scale Bars: 10 µm (A-E) and 2.5 µm (A’-E’’). (**F)** Quantification of the immunoreactivity of TrkB protein in EGFP positive neurons. (CTR, 1 ± 0.07; TrkB WT, 1.42 ± 0.08; TrkB SHC-, 1.73 ± 0.16; TrkB KD, 1.51 ± 0.13; n = 38-46 somata from 3 independent experiments). Numerical data are means ± SEM normalized to CTR. (**G)** Representative image of TrkB WT transduced neurons, stained for cleaved Caspase 3 (G’’, Cl.Csp3), MAP2 (G’’’), Hoechst (G’’’’), (**H)** Quantification of the percentage of Cl.Csp3 positive neurons in all EGFP positive neurons (TrkB WT, 0.38 ± 0.05; TrkB SHC-, 0.49 ± 0.05; TrkB PLC-, 0.44 ± 0.07; TrkB KD, 0.47 ± 0.07; n = 6-8 wells from 3 independent experiments). Numerical data are means ± SEM normalized to TrkB WT. Statistical significance was assessed by Kruskal-Wallis with Dunn’s multiple comparison test (*p < 0.05, **p < 0.01, ***p < 0.001; ns = not significant). For detailed statistical analysis refer to Table S3.


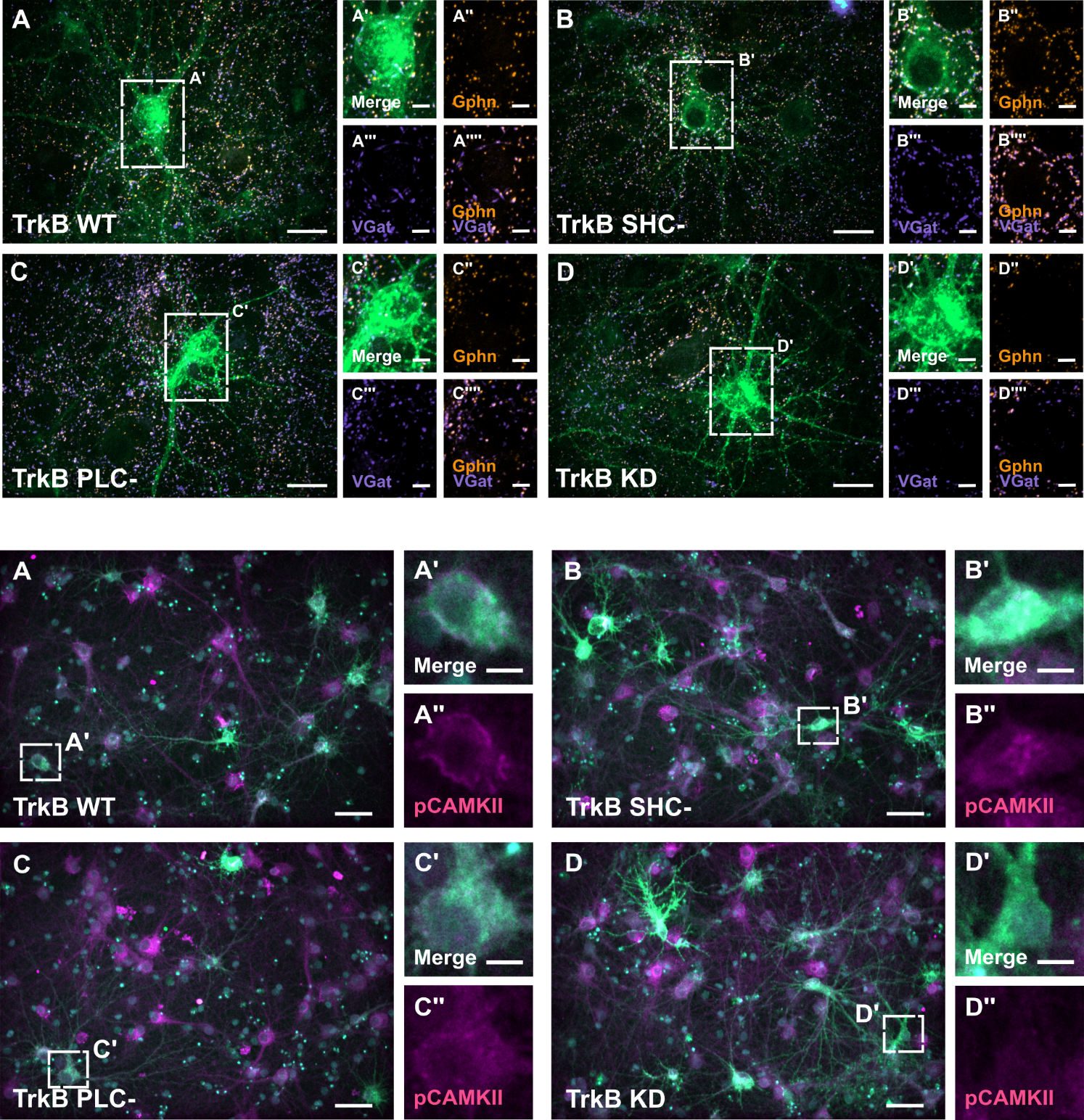


**Figure S5: Representative images of TrkB mutant overexpressing neurons stained for inhibitory pre- and postsynaptic marker. (A-D)** Representative images of primary hippocampal neurons at DIV14, overexpressing either EGFP-tagged TrkB WT (A) or mutants (B, C, D) stained for inhibitory pre and postsynaptic marker VGat and gephyrin (Gphn). The dashed rectangles indicate the respective enlarged sections depicted in A’, B’, C’ and D’. Gphn signals are shown in A’’, B’’, C’’ and D’’. VGat signals are shown in A’’’, B’’’, C’’’ and D’’’. Colocalized Gphn and VGat spots are shown in A’’’’, B’’’’, C’’’’ and D’’’’. Scale Bars: 10 µm (A-D) and 2.5 µm (A’-D’’’’).


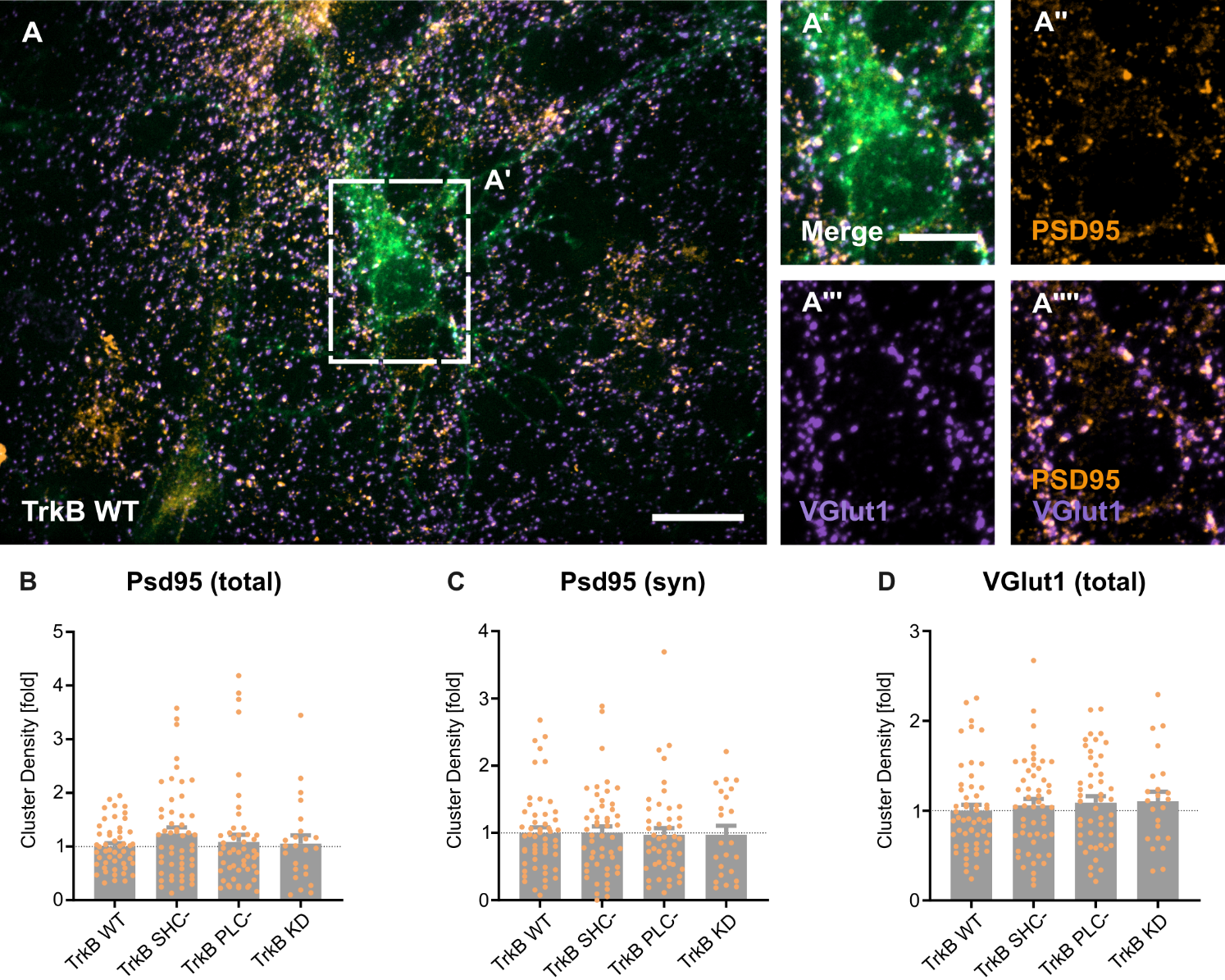


**Figure S6: TrkB mutants do not change excitatory synapse marker expression. (A)** Representative image of primary hippocampal neurons expressing TrkB WT. The dashed rectangle indicates the respective enlarged section depicted in A’. Signals for Psd95 and VGlut1 are shown in A’’ and A’’’, respectively. Colocalization of pre- and postsynaptic marker is depicted in A’’’’. Scale bars: 10 µm (A) and 5 µm (A’-A’’’’). (**B)** Quantification of total somatic Psd95 densities in EGFP-positive neurons (TrkB WT, 1 ± 0.06; TrkB SHC-, 1.24 ± 0.12; TrkB PLC-, 1.09 ± 0.13; TrkB KD, 1.05 ± 0.16; n = 24-51 somata from 3 independent experiments (2 independent experiments for TrkB KD). **(C)** Quantification of synaptic Psd95 densities (Psd95+VGlut1) in EGFP-positive neurons (TrkB WT, 1 ± 0.08; TrkB SHC-, 1.01 ± 0.09; TrkB PLC-, 0.97 ± 0.10; TrkB KD, 0,97 ± 0.13; n = 23-53 somata from 3 independent experiments (2 independent experiments for TrkB KD). **(D)** Quantification of somatic VGlut1-positive presynaptic terminals in EGFP-positive neurons (TrkB WT, 1 ± 0.06; TrkB SHC-, 1.06 ± 0.07; TrkB PLC-, 1.09 ± 0.07; TrkB KD, 1.10 ± 0.11; n = 23-54 somata from 3 independent experiments (2 independent experiments for TrkB KD)). Numerical data are means ± SEM and normalized to TrkB WT. Statistical significance was assessed by Kruskal-Wallis with Dunn’s multiple comparison test (**p < 0.01, nonsignificant comparisons are not noted). For detailed statistical analysis refer to Table S3.


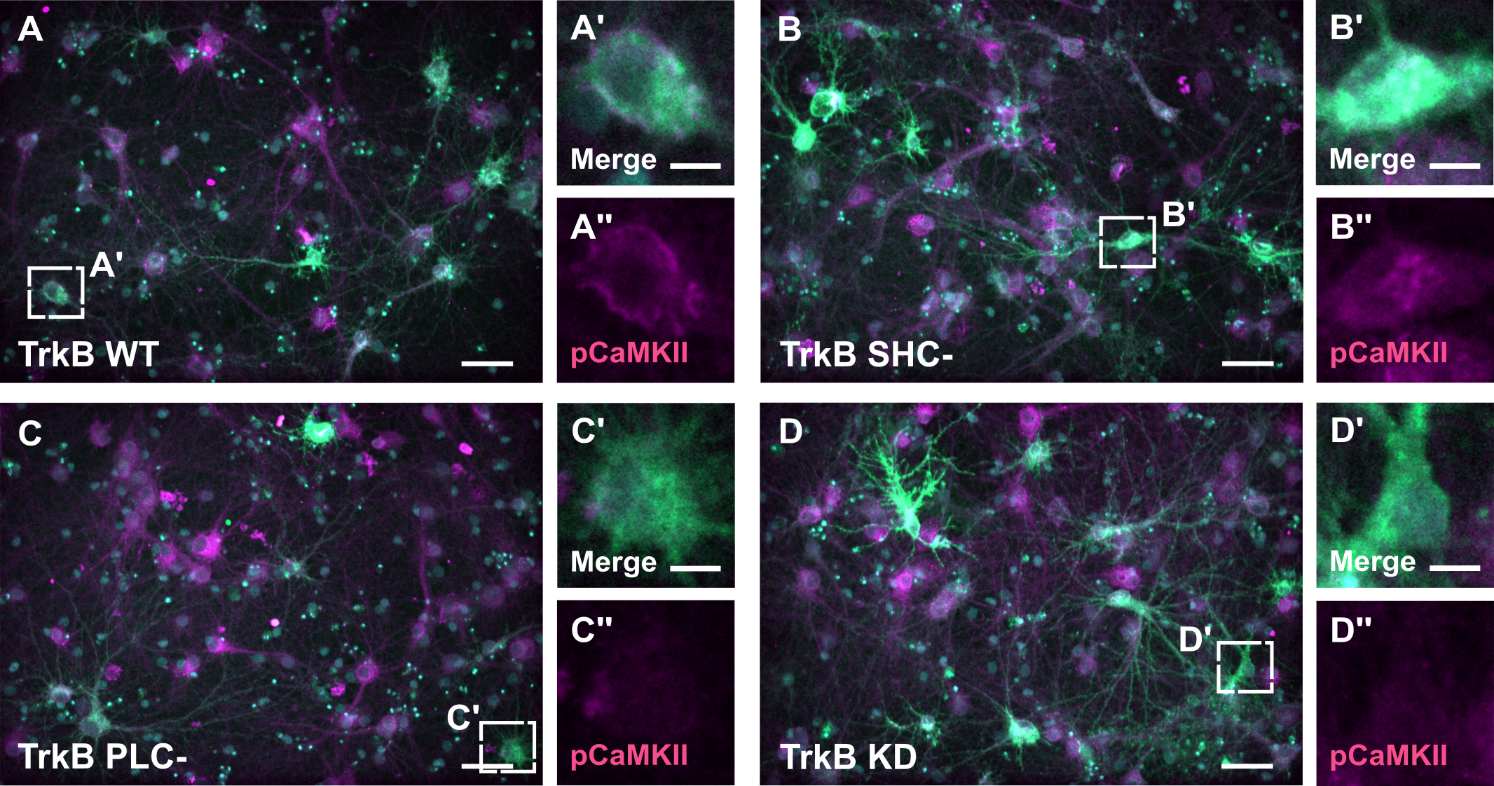


**Figure S7: Representative images of TrkB mutant overexpressing neurons stained for pCaMKII. (A-D)** Representative images of primary hippocampal neurons at DIV14, overexpressing either EGFP-tagged TrkB WT (A) or mutants (B, C, D), stained for activated CaMKII autophosphorylated at Thr286. The dashed rectangles indicate the enlarged sections depicted in A’, B’, C’, D’, respectively. pCaMKII signals are shown in A’’, B’’, C’’, D’’ and pan CaMKII in A’’’, B’’’, C’’’, D’’’. Scale Bars: 10 µm (A-D) and 2.5 µm (A’-D’’’’).


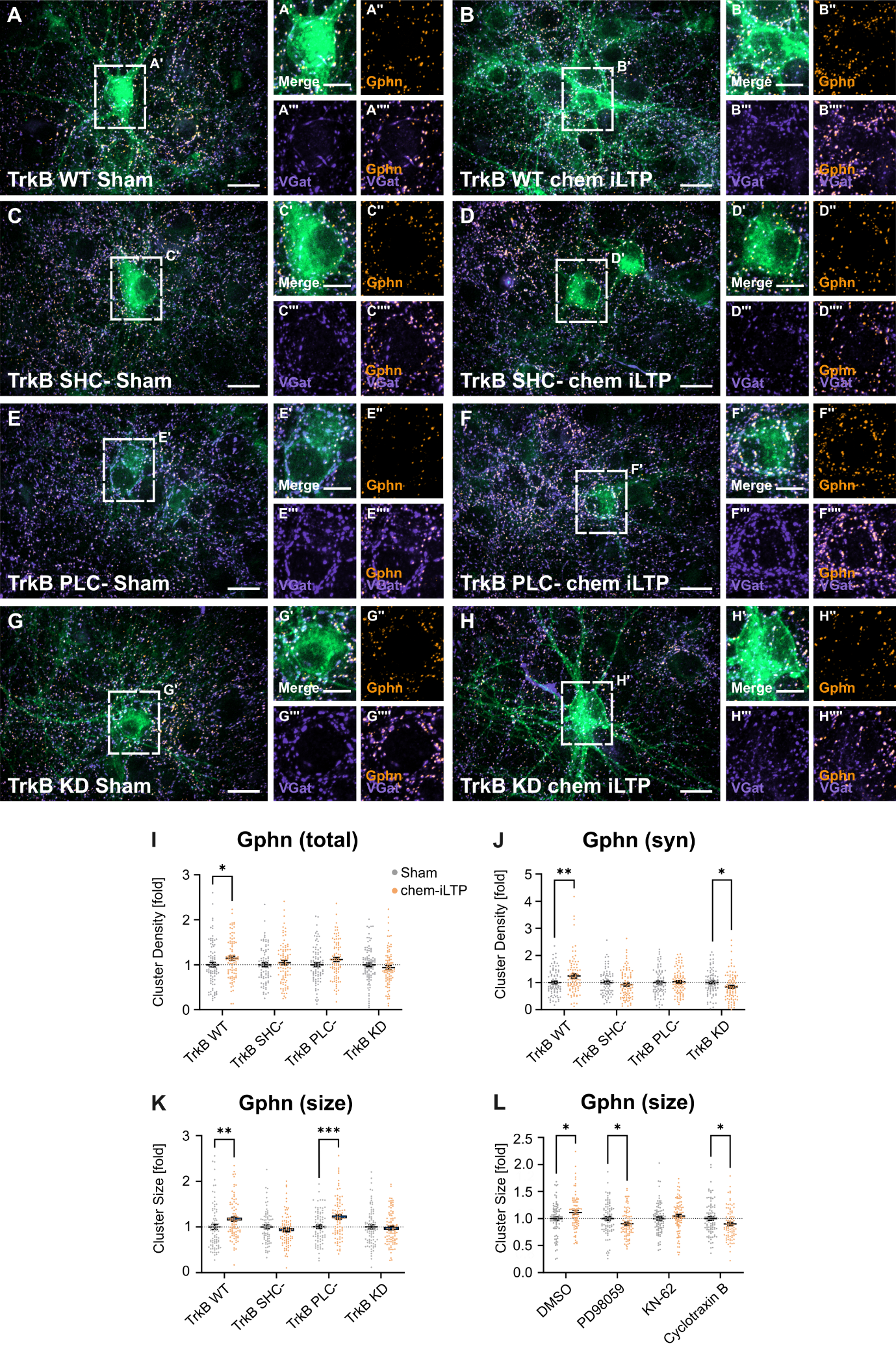


**Figure S8: Representative images of primary neurons under basal conditions and during chem iLTP. (A-H)** Representative images of primary hippocampal neurons, expressing the TrkB WT (A, B) or mutants (C-H) stimulated with sham solution (A, C, E, G) or NMDA+CNQX for chemical induction of iLTP (chem iLTP; B, D, F, H). The dashed rectangle implicates the enlarged section depicted in A’. B’, C’, D’, E’, F’, G’, H’, respectively. Neurons were stained for Gphn (A’’, B’’, C’’, D’’, E’’, F’’, G’’, H’’) and VGat (A’’’, B’’’, C’’’, D’’’, E’’’, F’’’, G’’’, H’’’). Colocalized Gphn and VGat spots are shown in A’’’’, B’’’’, C’’’’, D’’’’, E’’’’, F’’’’, G’’’’, H’’’’). Scale bars: 10 µm (A-H) and 5 µm (A’-H’’’’). **I** Total somatic gephyrin cluster densities. **J** Synaptic somatic gephyrin cluster densities. **K** Mean gephyrin cluster size. **L** Mean gephyrin cluster size. Numerical data are means ± SEM, obtained from three independent experiments, normalized to the respective untreated group. Statistical significance was assessed by 2way ANOVA with Tukey’s multiple comparison (p < 0.05, **p < 0.01, ***p < 0.001; nonsignificant comparisons are not noted). For detailed statistical values refer to Figure 4.

**
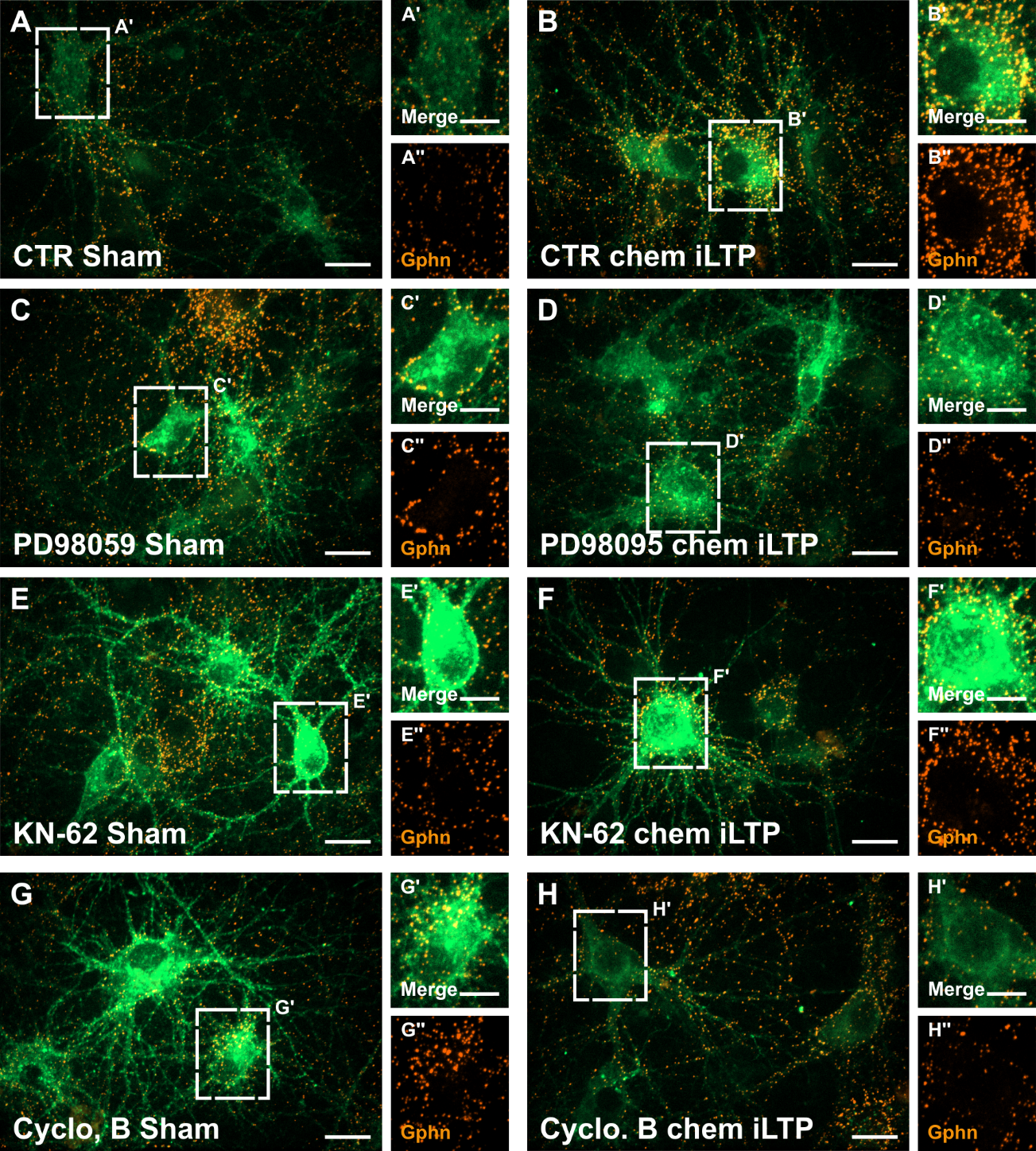
**

**Figure S9: Representative images of primary neurons under basal conditions and during chem iLTP in the presence of signaling inhibitors. A-H** Representative images of primary hippocampal neurons expressing TrkB WT stimulated with sham (A, C, E, G) or NMDA+CNQX for chemical induction of iLTP (chem iLTP; B, D, F, H) in the presence of DMSO (CTR; A, B) or inhibitors for MEK1 (PD98059; C, D), CaMKII (KN-62; E, F) or TrkB (Cyclotraxin B, Cyclo. B; G, H). Scale bars: 10 µm (A-H) and 5 µm (A’-H’’).

Table S1: Mutagenetic primers

| Mutant | Mutagenic primers (5'…3') |
| --- | --- |
| TrkB SHC- | CATTGAAAACCCCCAGTTCTTCGGTATCACCAAC |
| TrkB PLC- | GGCGTCGCCCGTCTTCCTGGACATCCTAG |
| TrkB KD | CTGGTGGCCGTGGCGACGCTGAAGGAC |

Table S2: miRNA Target Sequences

| miRNA | Target sequences (5'…3') |
| --- | --- |
| mi162 | TTAGGTTCCAACCTCGGAAAT |
| mi1973 | AGACCATGCCTGCTGCGATTT |

Table S3: Statistical data

| **Figure 1** | | |
| --- | --- | --- |
| **Comparison** | **n** | **p** |
| **Gphn proximal H(2) = 67.33, p < 0.0001** | | |
| miCTR vs. mi1973 | 79 vs. 95 | < 0.0001 |
| miCTR vs. mi162 | 79 vs. 96 | < 0.0001 |
| **Gphn distal H(2) = 3.75, p = 0.1532** | | |
| miCTR vs. mi1973 | 71 vs. 90 | 0.4602 |
| miCTR vs. mi162 | 71 vs. 90 | 0.1069 |
| **Gphn soma H(2) = 15.74, p 0.0004** | | |
| miCTR vs. mi1973 | 50 vs. 59 | 0.0022 |
| miCTR vs. mi162 | 50 vs. 57 | 0.0005 |
| **Gphn AIS H(2) = 2.29, p = 0.3190** | | |
| miCTR vs. mi1973 | 75 vs. 90 | 0.3356 |
| miCTR vs. mi162 | 75 vs. 78 | 0.4204 |
| **VGat F(2, 147) = 28.72, p < 0.0001** | | |
| miCTR vs. mi1973 | 50 vs. 50 | < 0.0001 |
| miCTR vs. mi162 | 50 vs. 50 | < 0.0001 |
| **Psd95 proximal H(2) = 0.062, p = 0.9696** | | |
| miCTR vs. mi1973 | 53 vs. 53 | > 0.9999 |
| miCTR vs. mi162 | 53 vs. 52 | > 0.9999 |
| **Figure 2** | | |
| **Gphn (total) H(3) = 20.76, p = 0.0001** | | |
| TrkB WT vs. SHC- | 45 vs. 55 | 0.0091 |
| TrkB WT vs. PLC- | 45 vs. 54 | > 0.9999 |
| TrkB WT vs. KD | 45 vs. 47 | > 0.9999 |
| **Gphn (syn) H(3) = 25.98, p < 0.0001** | | |
| TrkB WT vs. SHC- | 51 vs. 54 | > 0.9999 |
| TrkB WT vs. PLC- | 51 vs. 53 | 0.0028 |
| TrkB WT vs. KD | 51 vs. 44 | 0.0054 |
| **Gphn (size) H(3) = 11.09, p = 0.0113** | | |
| TrkB WT vs. SHC- | 49 vs. 60 | 0.0308 |
| TrkB WT vs. PLC- | 49 vs. 54 | 0.5553 |
| TrkB WT vs. KD | 49 vs. 43 | 0.0075 |
| **pCaMKII (Thr286) H(3) = 14.00, p = 0.0029** | | |
| TrkB WT vs. SHC- | 81 vs. 81 | 0.3887 |
| TrkB WT vs. PLC- | 81 vs. 79 | 0.0018 |
| TrkB WT vs. KD | 81 vs. 80 | 0.0133 |
| **Figure 3** | | |
| **AMP F(3, 75) = 2.710, p = 0.0510** | | |
| TrkB WT vs. SHC- | 21 vs. 21 | 0.3045 |
| TrkB WT vs. PLC- | 21 vs. 17 | 0.0167 |
| TrkB WT vs. KD | 21 vs. 20 | 0.5414 |
| **Event Frequency F(3, 74) = 4.683, p = 0.0047** | | |
| TrkB WT vs. SHC- | 21 vs. 20 | 0.9118 |
| TrkB WT vs. PLC- | 21 vs. 17 | 0.0197 |
| TrkB WT vs. KD | 21 vs. 20 | 0.9092 |
| **Figure 4** | | |
| **Gphn (total) F(1, 696) = 3.363, p = 0.0671** | | |
| TrkB WT sham vs. chem iLTP | 89 vs. 90 | 0.0273 |
| TrkB SHC- sham vs. chem iLTP | 80 vs. 90 | 0.4882 |
| TrkB PLC- sham vs. chem iLTP | 87 vs. 89 | 0.0815 |
| TrkB KD sham vs. chem iLTP | 90 vs. 89 | 0.3261 |
| **Gphn (syn) F(1, 697) = 0.06560, p = 0.7979** | | |
| TrkB WT sham vs. chem iLTP | 89 vs. 90 | 0.0016 |
| TrkB SHC- sham vs. chem iLTP | 80 vs. 90 | 0.3406 |
| TrkB PLC- sham vs. chem iLTP | 87 vs. 89 | 0.6974 |
| TrkB KD sham vs. chem iLTP | 90 vs. 90 | 0.0397 |
| **Gphn (size) F(1, 680) = 5.526, p = 0.0190** | | |
| TrkB WT sham vs. chem iLTP | 87 vs. 86 | 0.0070 |
| TrkB SHC- sham vs. chem iLTP | 78 vs. 88 | 0.3056 |
| TrkB PLC- sham vs. chem iLTP | 84 vs. 88 | 0.0006 |
| TrkB KD sham vs. chem iLTP | 87 vs. 90 | 0.7056 |
| **Gphn (size) F(1, 680) = 0.1375, p = 0.7109** | | |
| TrkB CTR sham vs. chem iLTP | 84 vs. 82 | 0.0129 |
| TrkB PD98059 sham vs. chem iLTP | 87 vs. 86 | 0.0259 |
| TrkB KN-62 sham vs. chem iLTP | 84 vs. 87 | 0.0381 |
| TrkB Cyclotraxin B sham vs. chem iLTP | 89 vs. 89 | 0.2948 |
| **Figure S3** | | |
| **TrkB Overexpression H(4) = 18.89, p = 0.0008** | | |
| CTR vs. TrkB WT | 41 vs. 46 | 0.0113 |
| CTR vs. TrkB SHC- | 41 vs. 45 | 0.0009 |
| CTR vs. TrkB PLC- | 41 vs. 42 | 0.0086 |
| CTR vs. TrkB KD | 41 vs. 38 | 0.0252 |
| TrkB WT vs. SHC- | 46 vs. 45 | > 0.9999 |
| TrkB WT vs. PLC- | 46 vs. 42 | > 0.9999 |
| TrkB WT vs. KD | 46 vs. 38 | > 0.9999 |
| TrkB SHC- vs. PLC- | 45 vs. 42 | > 0.9999 |
| TrkB SHC- vs. KD | 45 vs. 38 | > 0.9999 |
| TrkB PLC- vs. KD | 42 vs. 38 | > 0.9999 |
| **Cl. Csp3 H(3) = 1.994, p = 0.5737** | | |
| TrkB WT vs. SHC- | 8 vs. 8 | 0.5185 |
| TrkB WT vs. PLC- | 8 vs. 8 | > 0.9999 |
| TrkB WT vs. KD | 8 vs. 6 | > 0.9999 |
| **Figure S6** | | |
| **Psd95 (total) H(3) = 2.807, p = 0.4223** | | |
| TrkB WT vs. SHC- | 51 vs. 52 | > 0.9999 |
| TrkB WT vs. PLC- | 51 vs. 50 | > 0.9999 |
| TrkB WT vs. KD | 51 vs. 24 | > 0.9999 |
| **Psd95 (syn) H(3) = 0.2936, p = 0.9612** | | |
| TrkB WT vs. SHC- | 53 vs. 50 | > 0.9999 |
| TrkB WT vs. PLC- | 53 vs. 47 | > 0.9999 |
| TrkB WT vs. KD | 53 vs. 23 | > 0.9999 |
| **Psd95 (total) H(3) = 1.263, p = 0.7380** | | |
| TrkB WT vs. SHC- | 54 vs. 52 | > 0.9999 |
| TrkB WT vs. PLC- | 54 vs. 48 | 0.9656 |
| TrkB WT vs. KD | 54 vs. 23 | > 0.9999 |
